# Psychological therapy for children and young people with a diagnosis of autism or intellectual disabilities in mainstream child and adolescent mental health services: a cross-sectional survey of clinicians’ perspectives in England

**DOI:** 10.1136/bmjopen-2026-122190

**Published:** 2026-11-02

**Authors:** Naomi Williams, Helena Tuomainen, Julie Taylor, Jonathan Weiss, Kylie M Gray, Hayley L Trower, Parneet Pandher, Kayla Smith, Hayley Crawford

**Affiliations:** 1 University of Warwick Medical School, Coventry, Warwickshire, UK; 2 School of Nursing & Midwifery, University of Worcester, Worcester, UK; 3 Department of Nursing and Midwifery, University of Birmingham, Birmingham, UK; 4 Birmingham Women’s and Children’s Hospital NHS Foundation Trust, Birmingham, UK; 5 Department of Psychology, York University, Toronto, Ontario, Canada; 6 Intellectual Disabilities Research Institute, University of Birmingham—Edgbaston Campus, Birmingham, UK; 7 Health Sciences, University of Warwick, Coventry, UK; 8 Health Sciences, University of Warwick Medical School, Coventry, UK

**Keywords:** Adolescent, Child, Cross-Sectional Studies, Health Equity, Health Services, MENTAL HEALTH

## Abstract

**Abstract:**

**Objectives:**

To examine clinicians’ perspectives on delivering psychological therapy to autistic children and young people (CYP), CYP with intellectual disabilities and CYP with no autism or intellectual disabilities diagnoses in mainstream child and adolescent mental health services (CAMHS) in England. The study aimed to examine clinician-reported training, knowledge, confidence, skills, use of psychological therapies and adaptations and perceived barriers to access.

**Design:**

Cross-sectional observational survey study.

**Setting:**

Mainstream National Health Service (NHS) child and adolescent mental health services across 19 NHS Trusts in England.

**Participants:**

A total of 135 CAMHS clinicians participated. Eligible participants were healthcare professionals involved in delivering, supervising or leading psychological therapy for CYP aged 5–18 years within participating NHS Trusts. Clinicians completed survey sections relevant to their experience using built-in skip logic. Of the participants, 117 (86.7%) reported experience working with autistic CYP and 64 (47.4%) reported experience working with CYP with intellectual disabilities.

**Interventions:**

Not applicable.

**Primary and secondary outcome measures:**

Primary outcomes were clinicians’ self-reported knowledge, confidence and skills in delivering psychological therapy to autistic CYP and CYP with intellectual disabilities. Secondary outcomes included reported training, psychological therapies used, treatment adaptations, perceived evidence base of therapies and reported service access criteria.

**Results:**

Among clinicians with experience across the relevant CYP populations, clinicians reported lower knowledge, skills and confidence when delivering psychological therapy to CYP with intellectual disabilities, compared with autistic CYP and CYP with no autism or intellectual disabilities diagnoses. Self-reported preparedness was generally higher for autism than for intellectual disabilities. Seventy per cent of clinicians reported receiving autism-specific training compared with 47.6% for intellectual disabilities. Cognitive behavioural therapy was the most commonly reported psychological therapy, and making abstract concepts more concrete was the most frequently reported adaptation. Clinicians also reported greater uncertainty regarding the appropriateness of psychological therapies for CYP with intellectual disabilities.

**Conclusions:**

Clinicians working in mainstream CAMHS reported lower preparedness for delivering psychological therapy to CYP with intellectual disabilities than for autistic CYP and CYP without these diagnoses. The findings highlight the need for enhanced clinician training, greater implementation of evidence-informed adaptations and review of service access criteria to improve equitable access to psychological therapy. Future research should evaluate interventions that strengthen clinician capability and improve access to psychological therapies for CYP with intellectual disabilities.

**STRENGTHS AND LIMITATIONS OF THIS STUDY**

- *Allows study of multiple variables*: data were collected on multiple variables simultaneously (eg, demographics, three populations and various types of psychological therapies).
- *Useful for public health and policy planning*: the cross-sectional survey helped identify current needs, risk factors and target groups, which could inform decision-making and resource allocation.
- *Self-report bias*: Responses may be influenced by social desirability, memory errors or misunderstanding of questions, which can reduce validity.
- *Limited depth*: Closed questions can yield fixed response options, limiting participants’ ability to fully express their views and reducing context and nuance.

## Introduction

 In England in 2023, 20.3% of individuals aged 8–16 years were reported to have a probable mental health condition.^1^ Prevalence appears elevated among autistic children and young people (CYP) and CYP with intellectual disabilities: a meta-analysis estimated that approximately 28% of autistic CYP experience cooccurring mental health conditions,^2^ while around 40% of CYP with intellectual disabilities have a diagnosable mental health condition.^3^ Despite the heightened prevalence, autistic CYP and CYP with intellectual disabilities often may not have access to trained clinicians in mainstream child and adolescent mental health services (CAMHS) who can deliver appropriate psychological therapy.^4^

According to the DSM-5, autism is a neurodevelopmental condition characterised by differences in social communication and interaction, alongside restricted and repetitive patterns of behaviour, interests or activities.^5^ The International Classification of Diseases, 11th Revision (ICD-11) describes intellectual disabilities as characterised by significant limitations in intellectual functioning and adaptive behaviour, with onset during the developmental period.^6^

Existing literature highlights gaps in clinicians’ knowledge, confidence and implementation of adaptations, particularly in mainstream CAMHS; the evidence base and volume of work differ when working with autistic CYP and CYP with intellectual disabilities, so findings should be considered separately for each group.^7 8^

Previous research has identified several barriers to accessing psychological therapy for autistic CYP and CYP with intellectual disabilities. These include systemic factors, such as organisational barriers, inflexible eligibility thresholds, insufficient availability of services and the suboptimal quality of existing services.^9^ At the clinician level, barriers include gaps in clinicians’ knowledge and a reluctance to adapt psychological therapy to accommodate specific needs.^10^ Knowledge gaps can lead professionals to conflate mental health conditions with a neurodevelopmental diagnosis.^11 12^ Such barriers include mental health conditions not being recognised by clinicians during triage and as a result they are not allocated to a clinician for a psychological therapy.^13^ This can result in untreated mental health conditions and ultimately lead to a mental health crisis and an increase in inpatient admissions. Other inequalities, including socioeconomic status and ethnicity, compound this issue.^14^

Other barriers to care may stem from clinicians’ attitudes and beliefs, for example, a study conducted in the USA revealed that clinicians perceived the provision of psychological therapy to autistic CYP as a frustrating process.^15^ Canadian clinicians reported feeling less knowledgeable and confident when supporting autistic CYP compared with individuals with attention deficit hyperactivity disorder (ADHD^16^). Identifying clinician challenges with knowledge, skills and attitudes can lead to improvements in focused training initiatives in identifying, understanding and appropriately accommodating autistic CYP and CYP with intellectual disabilities.^17^

The National Institute for Health Care and Excellence guidelines emphasise that CAMHS clinicians must adapt psychological therapy and provide reasonable adjustments at the service level to meet the specific needs of autistic CYP and CYP with intellectual disabilities, such as the use of visual supports, shortened language and concrete examples.^18^ A recent systematic review and meta-analysis found that providing adaptations to psychological therapy and implementing reasonable adjustments, including environmental changes and caregiver involvement, can improve accessibility and acceptability for autistic CYP.^19^ Another recent systematic review highlighted that adaptations to psychological therapy, particularly cognitive behavioural approaches, are commonly required when working with people with intellectual disabilities, including multisensory methods, activities, communication, delivery medium, additional support and structure.^20^ However, the strength and consistency of evidence around adaptations remains variable.^20^ Some evidence also suggests that adaptations to evidence-based therapies, such as cognitive behavioural therapy, may benefit autistic CYP^21 22^; however, research gaps exist.

Creative therapies, including art, music, drama, LEGO, outdoor activities and animal-assisted therapies, show promising evidence as psychological treatments for autistic CYP and CYP with intellectual disabilities.^23 24^ However, there remains scarce research regarding their overall impact.^25^ By critically reflecting on the intersecting aspects of an individual’s identity,^14^ as well as cooccurring neurodevelopmental conditions such as autism, ADHD and intellectual disabilities,^26^ clinicians can implement culturally competent approaches.^27 28^

Psychological treatment refers to structured interventions delivered by mental health professionals that seek to influence cognitive, emotional, behavioural or interpersonal processes to improve health or functioning.^29^ These approaches are informed by psychological theory and evidence regarding processes of change and can be delivered by various healthcare professionals in mainstream CAMHS.^29^

It is unclear what psychological therapies are offered to autistic CYP and CYP with intellectual disabilities in mainstream CAMHS in England, and to what extent clinicians adapt psychological therapy for this population. It is also unclear whether clinicians have been specifically trained to deliver psychological therapy for this population. Surveying a diverse range of clinicians who provide psychological therapy to both groups of CYP within the same study enables comparison of clinician training, confidence, reported adaptations, and perceived access criteria, helping to identify areas where workforce development may be most needed.

The current study aimed to explore clinicians’ perspectives on psychological therapy for autistic CYP and CYP with intellectual disabilities in mainstream CAMHS across England. In addition, clinicians’ self-reported knowledge, confidence and skills were compared across CYP with no autism or intellectual disabilities diagnoses, autistic CYP and CYP with intellectual disabilities.

The following research questions were posed:

1. Have clinicians received training specific to delivering psychological therapy to autistic individuals and individuals with intellectual disabilities?
2. What adaptations do clinicians report using when delivering psychological therapy for working with autistic individuals and individuals with intellectual disabilities?
3. What are clinicians’ knowledge areas and confidence levels when providing psychological therapy to individuals with no autism or intellectual disabilities diagnoses, autistic individuals and individuals with intellectual disabilities?
4. Do clinicians feel that they have the skills to deliver psychological therapy to individuals with no autism or intellectual disabilities diagnoses, autistic individuals and individuals with intellectual disabilities?
5. Which psychological therapy do clinicians believe to be evidence-based for supporting autistic individuals and individuals with intellectual disabilities?
6. What do clinicians report about barriers to access for autistic individuals and individuals with intellectual disabilities?

## Methods

This study was an observational, cross-sectional survey of clinicians working in mainstream CAMHS across England. The study aimed to examine clinicians’ perspectives on delivering psychological therapy to autistic CYP and CYP with intellectual disabilities, including clinicians’ reported training, knowledge, confidence, skills, treatment approaches and adaptations and perceived barriers to accessing psychological therapy for these populations.

### Patient and public involvement

The study was appraised by PPI Teams across England to improve the design of the survey and to review the recruitment flyer, protocol, consent form and participant information sheet.

The patient and public involvement (PPI) contributors included parents/caregivers (n=14), autistic CYP (n=7) and CYP with intellectual disabilities (n=3), across four PPI groups.

Recruitment of PPI members took place through social media, local charities, churches, community groups and parent partnership groups. The process included amending the language and adding UK-relevant psychological therapies and questions on triage, assessment, recruitment, training and decision-making.

### Recruitment

A sample of mainstream CAMHS clinicians was recruited from 19 NHS Trusts across England using purposive sampling to capture variation in geography, professions and service types.^30^ Eligible participants were professionals involved in delivering psychological therapy, directly or in a supervised capacity, to CYP aged 5–18 years across participating NHS Trusts. Some participating professionals did not routinely deliver formal psychological therapies; however, they may have contributed within multi-disciplinary child and adolescent mental health teams, as a supervisor or team lead. Therefore, the findings are shared from clinicians’ self-reports of psychological therapy delivery and adaptations (including service-level reasonable adjustments), interpreted considering role diversity.^29^

Variation in geography, profession and service context across NHS Trusts was sought.^31^ Study information packs were provided to all NHS Trust research and development teams who decided on their own process for informing local CAMHS about the survey. Interested individuals were invited to a remote drop-in session via Microsoft Teams, which allowed them to ask questions and hear more information about the study. Administration leads shared an online Microsoft Forms survey link and recruitment flyer via email to eligible professionals.

### Sample size

No formal sample size calculation was undertaken because this was an exploratory cross-sectional survey. We aimed to recruit as many eligible clinicians as possible across participating NHS Trusts during the study period. Recruitment invitations were disseminated to participating NHS Trusts in accordance with local procedures. As recruitment was managed locally, the research team did not have access to the total number of clinicians who received the invitation; therefore, an overall response rate could not be calculated.

### Procedure

All participants provided informed consent by completing an online consent form before completing the survey. Each participant completed the same survey, which took on average 12 min to complete online. At the end of the survey, participants could consent to be entered into a prize draw for £100 or £50 for their participation.

### Measures

#### Sociodemographic characteristics

Participants provided sociodemographic characteristics (eg, age, gender, ethnicity, employment status, professional registration) and details about their professional background (eg, primary role, number of individuals on their current caseload, how often they have contact with autistic CYP and CYP with intellectual disabilities, number of years they have been in professional practice).

The primary outcome measures were clinicians’ self-reported knowledge, confidence and skills in delivering psychological therapy to autistic CYP and CYP with intellectual disabilities. Secondary outcome measures included clinicians’ reported training, psychological therapy approaches, treatment adaptations, perceived evidence base of psychological therapies and service access criteria.

#### Online mental health provider survey

The online mental health provider survey used in this study (see online supplemental information for the survey instrument) was adapted from an instrument developed in Canada and the USA.^16 32^ The adapted survey instrument was not formally pilot tested prior to dissemination. However, it was developed from previously published survey instruments with permission from the original authors and refined through consultation with CAMHS clinicians and PPI contributors, to improve clarity, relevance, acceptability and applicability to the UK CAMHS context.

While the original survey focused on autism and ADHD, the current study targeted autistic CYP and CYP with intellectual disabilities to address the knowledge gap and the need for further investigation of the quality of mental healthcare, specifically among CYP with intellectual disabilities in England. Permission was obtained from the original survey authors to make minor modifications to the language and role titles to reflect mental health services in England.^16 32^ Clinicians completed only the survey sections relevant to their current caseload and previous clinical experience within mainstream CAMHS.^16 32^ The survey incorporated a built-in skip logic, so that participants were automatically routed to relevant questions based on their previous responses (eg, clinicians without experience delivering psychological therapy to CYP with intellectual disabilities were not presented with those items).

All participants (n=135) completed questions relating to CYP without autism or intellectual disabilities. Clinicians who reported relevant experience also completed additional survey sections relating to autistic CYP (n=117) and/or CYP with intellectual disabilities (n=64). Consequently, participants completed only the survey sections applicable to their professional experience.

The survey included measures relating to delivering psychological therapy to autistic CYP, CYP with intellectual disabilities and CYP with no autism or intellectual disabilities diagnoses. Some measures assessed self-reports on delivering psychological therapy to autistic CYP and CYP with intellectual disabilities separately, while the general knowledge, confidence and skills items were completed for all three CYP populations to enable within-clinician comparisons.

The survey consisted of eight domains:

1. Treatment approaches: Participants were asked to select treatment approaches used from a list of 20 (eg, cognitive behavioural therapy, emotion-focused therapy, acceptance and commitment therapy).
2. Evidence-based: Of the selected treatment approaches, participants were asked to indicate which treatment approaches they believed to be evidence-based.
3. Treatment adaptations: Participants were asked to select any treatment adaptations (eg, making abstract concepts more concrete) to psychological therapy that were made in the past.
4. Confidence: Participants completed the Therapist Confidence Scale originally developed for clinicians working with CYP with intellectual disabilities,^33^ and adapted in the present study to include use with autistic CYP. The item wording was adjusted to refer separately to autistic CYP and to people with intellectual disabilities; the item content was otherwise unchanged. For clarity, these adapted versions are referred to as the Therapy Confidence Scale-Intellectual Disabilities-Autism (TCS-ASD) and Therapy Confidence Scale-Intellectual Disabilities (TCS-ID), respectively (TCS-ASD/ID). A 14-item measure that asks clinicians to rate their confidence in using well-known principles during a therapeutic relationship (eg, developing empathy, communication, assessment, ending therapy). The TCS-ASD/ID has demonstrated strong reliability and a positive association with clinicians’ extent of training in developing a rapport with this population.^33^
  **Additionally**, participants answered **general confidence items** (eg, I feel that I have the *confidence to deliver psychological therapy to children **who are not** *autistic or **who do not have an** *intellectual disability/ *autistic children/young people/ *children/young people with an **intellectual disability**). These items were used in the within-clinician comparisons reported in the results.
5. Knowledge: Participants completed a six-item measure^15^ to rate their knowledge on core symptoms, cooccurring problems, impact of characteristics on behaviour, developing a treatment plan, delivering treatment and identifying progress toward treatment goals using a five-point scale. Internal consistency for the knowledge scale has previously been reported as good.^15 34^
  **Additionally**, participants answered **general knowledge items** (eg, I feel that I have the *knowledge to deliver psychological therapy to children **who are not** *autistic or **who do not have an** *intellectual disability/ *autistic children/young people/ *children/young people with an **intellectual disability**). These items were used in the within-clinician comparisons reported in the results.
6. Skills: Participants **answered general skills items** (eg, I feel that I have the *skills to deliver psychological therapy to children **who are not** *autistic or **who do not have an** *intellectual disability/ *autistic children/young people/ *children/young people with an **intellectual disability**). These items assessed perceived skills across the same three CYP populations and were used in the within-clinician comparisons reported in the results.
7. Training: Participants were asked to report any training experiences, responsibilities for triaging and assessing individuals, and involvement in referral allocation, pathway planning, decisions, training and recruitment.
8. Criteria: Participants were asked if their service had established access criteria (eg, exclusionary criteria, practices and policies).

In this study, *adaptations* refer primarily to adaptations made during the delivery of psychological therapy; however, clinicians were also asked about reported reasonable adjustments, criteria and processes at referral and triage that may influence access. The mental health provider survey used the term *psychological therapy*; this wording is retained when referring to specific questionnaire items.

#### Data analysis

All data were analysed using SPSS 29.0.2.0. Descriptive statistics and frequency tables were used to summarise survey responses and distribution frequencies.

Wilcoxon signed-rank test and Friedman test were used because the survey responses were measured on ordinal Likert-type scales and involved paired within-clinician comparisons. For any test, a p value less than 0.05 indicates statistical significance. All p values were two-sided.

For the six-item knowledge measure, Wilcoxon signed-rank tests were used to compare clinicians’ self-reported knowledge for working with autistic CYP versus CYP with intellectual disabilities; because six knowledge items were compared, Bonferroni-adjusted significance thresholds were considered when interpreting the results to reduce risk of type I error. Only clinicians with experience with both autistic CYP and CYP with intellectual disabilities were included in this analysis. The effect size r for a Wilcoxon signed-rank test was computed by dividing the absolute value of the standardised test statistic (z) by the square root of the total number of pairs (N), using the formula $r=\frac{\left|z\right|}{\sqrt{N}}$. The effect size r classifies the strength of the relationship into small (r=0.10), medium (r=0.30) and large (r=0.50) effects.

For the general knowledge, confidence and skills items, Friedman tests were used to compare clinicians’ ratings of clinicians’ knowledge, confidence and skills across three CYP populations (ie, autistic CYP, CYP with intellectual disabilities and CYP with no autism or intellectual disabilities diagnoses). Only clinicians with experience with all three CYP populations were included in this analysis. When the Friedman test yielded significant results, post hoc pairwise comparisons using Wilcoxon signed-rank test with p values adjusted using the Bonferroni method were conducted to locate the specific differences in clinician knowledge, confidence or skills across CYP populations. The most commonly used effect size for the overall Friedman test is Kendall’s W (coefficient of concordance). The effect size W classifies the strength of the relationship into small (r=0.10), medium (r=0.30) and large (r=0.50) effects. W can be computed using the following formula: $W=\frac{\chi^{2}}{N(k-1)}$, where $\chi^{2}$ is the chi-square test statistic from the Friedman test, N is the total sample size (ie, number of subjects) and k is the number of repeated measurements per subject. P values were adjusted via Bonferroni correction to maintain a global significance level of 0.05.

## Results

A total of 135 clinicians from 19 NHS Trusts across England responded, although response rates varied across survey sections.

Of clinicians surveyed, 117 (86.7%) confirmed that they had experience of delivering psychological therapy to autistic CYP, and 64 (47.4%) reported experience delivering psychological therapy to CYP with intellectual disabilities, and all 135 clinicians responded to items relating to CYP with no autism or intellectual disabilities diagnoses, accounting for a full sample. Lower response rates for items related to CYP with intellectual disabilities reflected the survey’s built-in skip logic, which presented these questions only to clinicians reporting relevant clinical experience. Therefore, these differences represent the planned survey design rather than missing data.

### Demographic information

Participants (n=135) responded to the survey items about their previous and/or current experiences of working in mainstream CAMHS even if they were not currently based in those services.

Participants included 135 clinicians (mean age=39 years, SD=10.03) across England. Most participants were service professionals with direct clinical contact (98.5%), with over one-third (37.8%) having supervisory responsibilities. Additionally, over one-half of the clinicians were registered with the Health Care Professions Council (31.1%) or the Nursing & Midwifery Council (21.5%); however, some clinicians did not hold a professional membership (16.3%), as the survey included responses from educational mental health practitioners employed by mainstream CAMHS (ie, mental health support teams that are based in schools, sixth forms and colleges). Demographic information is provided in table 1.

**Table 1:** Demographic characteristics of NHS clinicians working in mainstream child and adolescent mental health services (n=135)

Characteristic	n=135 (%)
Gender	
Female	122 (90.3%)
Male	12 (8.8%)
Prefer not to say	1 (0.7%)
Ethnicity	
Asian	6 (4.4%)
Black	4 (2.9%)
White	121 (89.6%)
Mixed	3 (2.2%)
Prefer not to say	1 (0.7%)
Service	
Community	90 (66.6%)
Forensic Unit	4 (2.9%)
Mental Health Support Team	24 (17.7%)
Neurodevelopmental Service	15 (11.1%)
Research	2 (1.4%)
Profession	
Creative therapist	6 (4.4%)
Cognitive behavioural therapist	13 (9.6%)
Clinical psychologist	25 (18.5%)
Speech, Language and occupational therapist	8 (5.9%)
Psychiatrist	6 (4.4%)
Psychotherapist	10 (7.4%)
Registered nurse	39 (28.8%)
Social worker	8 (5.9%)
Well-being practitioner	8 (5.9%)
Educational mental health practitioner	12 (8.8%)
Professional membership	
Health Care Professions Council	42 (31.1%)
Nursing & Midwifery Council	29 (21.5%)
British Association for Behaviour and Cognitive Psychotherapy	13 (9.6%)
Social Work England	11 (8.1%)
General Medical Council	5 (3.7%)
Association of Child Psychotherapists	3 (2.2%)
British Psychological Society	3 (2.2%)
British Association for Counselling and Psychotherapy	2 (1.5%)
British Association of Play Therapists	2 (1.5%)
United Kingdom Council for Psychotherapy	2 (1.5%)
Member Royal College of Psychiatrists	1 (0.7%)
None	22 (16.3%)
Years of practice	
0–2	34 (25.1%)
3–5	28 (20.7%)
6–9	24 (17.7%)
10–14	20 (14.8%)
15–19	11 (8.1%)
20+	18 (13.3%)
Region	
Midlands	75 (55.6%)
East of England	23 (17%)
North East and Yorkshire	23 (17%)
London	7 (5.2%)
South East	5 (3.7%)
North West	2 (1.5%)

Item non-response reflected planned skip patterns within the survey design, rather than missing data. For questions about the overview of service provision, training, treatment adaptations, knowledge areas and confidence levels and skills, only clinicians indicating relevant experience were routed to these items. In total, 117 clinicians (86.7%) responded about receiving training to deliver psychological therapy to autistic CYP and 64 clinicians (47.4%) responded regarding people with intellectual disabilities.

To compare clinicians supporting autistic CYP against those supporting CYP with intellectual disabilities, only professionals with experience across both CYP populations (n=64) were included in the subsequent analyses for reported likelihood of delivering psychological therapy, training, treatment adaptations, knowledge, confidence, skills, and treatment approaches and perceived evidence base. For access criteria, all clinicians (n=135) were included in the analysis.

### Reported likelihood of delivering psychological therapy by clinicians who work with children and young people and appropriateness of psychological therapy for children and young people

Among clinicians (n=64) with experience of delivering psychological therapy to both autistic CYP and CYP with intellectual disabilities, the following results on the likelihood of delivering psychological therapy and their views on the appropriateness of the psychological therapy for these populations are reported in this section. Clinicians reported a lower likelihood of delivering psychological therapy to CYP with intellectual disabilities than to autistic CYP.

As shown in table 2, 54.7% (n=35) of clinicians reported that they were extremely likely to deliver psychological therapy for autistic CYP; however, 36.5% (n=23) reported that they were uncertain whether the chosen psychological therapy was appropriate. 14.1% (n=9) reported that they were extremely likely to deliver psychological therapy for CYP with intellectual disabilities; however, 43.8% (n=28) were uncertain whether the chosen psychological therapy was appropriate.

**Table 2:** Clinician report on the likelihood of delivering psychological therapy and appropriateness of chosen approach for autistic children and young people and children and young people with intellectual disabilities

Likelihood of delivering psychological therapy by clinicians who works with children and young people	For autistic children and young people (n=64) n (%)	For children and young people with intellectual disabilities (n=64) n (%)
Extremely likely	35 (54.7%)	9 (14.1%)
Quite likely	16 (25.0%)	17 (26.6%)
Slightly likely	4 (6.3%)	16 (25.0%)
Neither (or not sure)	1 (1.6%)	3 (4.7%)
Slightly unlikely	3 (4.7%)	7 (10.9%)
Quite unlikely	2 (3.1%)	7 (10.9%)
Extremely unlikely	3 (4.7%)	5 (7.8%)
Appropriateness of psychological therapy for children and young people		
Very sure appropriate	4 (6.3%)	6 (9.4%)
Sure appropriate	31 (49.2%)	26 (40.6%)
Uncertain	23 (36.5%)	28 (43.8%)
Not appropriate	5 (10.0%)	4 (6.3%)

### Training

Question one*: have clinicians received training specific to delivering psychological therapy to autistic individuals and individuals with intellectual disabilities?*

Of those clinicians with experience across the two CYP populations (autistic CYP and CYP with intellectual disabilities), 81.3% of clinicians (52 out of 64) reported receiving training to deliver psychological therapy to autistic CYP compared with only 46.9% (30 out of 64) to CYP with intellectual disabilities.

### Treatment adaptations

Question two*: what adaptations do clinicians report using when delivering psychological therapy for working with autistic individuals and individuals with intellectual disabilities?*

Among clinicians (n=64) with experience delivering psychological therapy to both autistic CYP and CYP with intellectual disabilities, the following results describe reported adaptations used during psychological therapy for these populations.

As shown in table 3, adaptations used varied, among the most frequently used adaptations were ‘making abstract concepts more concrete’ (autism: 78.6%, intellectual disabilities: 68.8%).

**Table 3:** Clinician self-report of treatment adaptations used when delivering psychological therapy for autistic children and young people and children and young people with intellectual disabilities

Adaptations used during psychological therapy	Autistic children and young people (n=64), n (%)	Children and young people with intellectual disabilities (n=64), n (%)
Make abstract concepts more concrete	47 (73.4%)	44 (68.8%)
Provide detailed information	2 (3.1%)	4 (6.3%)
Provide structure and predictability	10 (15.6%)	7 (10.9%)
Support with processing verbal information	2 (3.1%)	1 (1.6%)
Capitalise on strengths	1 (1.6%)	2 (3.1%)
Minimise sensory distraction	0 (0%)	2 (3.1%)
Length of sessions: shorter	1 (1.6%)	1 (1.6%)

Note. Clinicians could select multiple options. Percentages may not sum to 100% due to rounding.

### Clinician knowledge and confidence

Question three*: what are clinicians’ knowledge areas and confidence levels when providing psychological therapy to individuals with no autism or intellectual disabilities diagnoses, autistic individuals and individuals with intellectual disabilities?*

### Knowledge

The following analyses are based on clinicians with experience delivering psychological therapy to both autistic CYP and CYP with intellectual disabilities (n=64). Wilcoxon signed-rank tests were used to compare self-reported knowledge across the two populations.

Wilcoxon signed-rank tests were used to compare clinicians’ self-reported knowledge for working with autistic CYP versus CYP with intellectual disabilities (table 4). Only clinicians with experience working with both autistic CYP and CYP with intellectual disabilities were included in this analysis. Clinicians had statistically significantly better knowledge when delivering psychological therapy to autistic CYP than to CYP with intellectual disabilities in terms of cooccurring conditions (mean scores: 2.81 vs 2.16; p<0.001), core symptoms (mean scores: 3.02 vs 2.23; p<0.001), delivering therapy (mean scores: 2.41 vs 1.94; p<0.001), developing therapy (mean scores: 2.31 vs 1.86; p<0.001), identifying progress (mean scores: 2.38 vs 2.06; p=0.011) and impact on mental health (mean scores: 2.86 vs 2.25; p<0.001).

**Table 4:** Clinician self-rated knowledge for delivering psychological therapy to autistic children and young people and children and young people with intellectual disabilities

	Autistic children and young people		Children and young people with intellectual disabilities		Mean difference (M (SD))	Wilcoxon signed-rank test
	M (SD)	Mdn (IQR)	M (SD)	Mdn (IQR)		
Cooccurring conditions	2.81 (0.85)	3 (1)	2.16 (0.96)	2 (1.75)	0.66 (0.86)	W=19, SE=51.79, z=−4.731, p<0.001, r=0.59
Core symptoms	3.02 (0.81)	3 (2)	2.23 (1.02)	2 (2)	0.78 (0.95)	W=48, SE=72.09, z=5.021, p<0.001, r=0.63
Delivering therapy	2.41 (0.95)	2 (1)	1.94 (1.14)	2 (2)	0.47 (0.94)	W=142, SE=65.09, z=−3.511, p<0.001, r=0.44
Developing therapy	2.31 (1.02)	2 (1)	1.86 (1.10)	2 (2)	0.45 (0.85)	W=137, SE=64.25, z=−3.634, p<0.001, r=0.45
Identifying progress	2.38 (1.00)	2 (1)	2.06 (1.10)	2 (2)	0.31 (0.92)	W=196, SE=61.41, z=−2.532, p=0.01, r=0.32
Impact on mental health	2.86 (0.85)	3 (1)	2.25 (0.96)	2 (1)	0.61 (0.90)	W=67.5, SE=63.31, z=−4.486, p<0.001, r=0.56

Note. Only clinicians with experiences with both patient populations (autistic children and young people (CYP) and CYP with intellectual disabilities) were included in this analysis. Results of Shapiro-Wilk test (p<0.05) suggested that the data (difference between autistic CYP and CYP with intellectual disabilities) were not normally distributed for the six knowledge measures.

IQR, IQR range; M, mean; Mdn, median; p, p value; r, effect size of Wilcoxon signed-rank test; SD, SD deviation; SE, SE error; W, Wilcoxon signed-rank test statistic; z, standardised test statistic.

Among clinicians (n=64) with experience across all three CYP populations (CYP with no autism or intellectual disabilities diagnoses, autistic CYP and CYP with intellectual disabilities), a Friedman test was used to compare self-reported knowledge of delivering psychological therapy across the three populations. Clinicians rated their knowledge level about delivering psychological therapy to CYP with no autism or intellectual disabilities diagnoses (M=2.69, SD=0.83; Mdn=3, IQR=1), autistic CYP (M=2.34, SD=0.82; Mdn=2, IQR=1) and CYP with intellectual disabilities (M=1.78, SD=1.00; Mdn=2, IQR=1). A Friedman test revealed a significant difference in self-perceived knowledge level about delivering psychological therapy to CYP with no autism or intellectual disabilities diagnoses, autistic CYP and CYP with intellectual disabilities (χ^2^(2)=45.676, p<0.001, Kendall’s W=0.357). Post hoc comparison tests using the Wilcoxon signed-rank test indicated clinicians felt statistically significantly less knowledgeable about delivering psychological therapy to CYP with intellectual disabilities than to CYP with no autism or intellectual disabilities diagnoses (p<0.001, r=0.680) and autistic CYP (p=0.001, r=0.464). There was no statistically significant difference in knowledge level about delivering psychological therapy to CYP with no autism or intellectual disabilities diagnoses and autistic CYP (p=0.254, r=0.216).

### Confidence

The following results are based on clinicians with experience delivering psychological therapy to both autistic CYP and CYP with intellectual disabilities (n=64). Descriptive statistics are presented for clinicians’ self-reported confidence across the two populations.

As shown in table 5, clinicians reported moderate to high levels of confidence when delivering psychological therapy to autistic CYP (means ranged from 2.70 to 3.56) and CYP with intellectual disabilities (means ranged from 2.29 to 3.28). Confidence ratings were consistently higher for autistic CYP than for CYP with intellectual disabilities. Overall, among clinicians with experience across the relevant CYP populations, clinicians reported greater confidence in core interpersonal skills such as empathy and listening, than assessment and intervention related competencies. Confidence was generally lower when working with CYP with intellectual disabilities compared with autistic CYP.

**Table 5:** Clinician reports of confidence for delivering psychological therapy to autistic children and young people and children and young people with intellectual disabilities

Items—Therapist Confidence Scale—Autism/intellectual disabilities Confidence in ability to:	Autistic children and young people M (SD) (n=64)	Children and young people with intellectual disabilities M (SD) (n=64)
Listen carefully	3.45 (0.64)	3.02 (0.92)
Be empathetic	3.56 (0.58)	3.28 (0.88)
Understand special issues	3.27 (0.67)	2.84 (0.99)
Communicate	3.13 (0.70)	2.87 (0.85)
Develop a therapeutic relationship	3.20 (0.74)	2.98 (0.85)
Gather information	3.13 (0.75)	2.70 (0.91)
Use assessments	2.78 (0.86)	2.35 (1.00)
Explain results of an assessment process	2.80 (0.88)	2.29 (0.97)
Use knowledge about mental health difficulties in formulating the needs	3.03 (0.85)	2.63 (0.99)
Help to identify issues	2.86 (0.81)	2.43 (1.03)
Use knowledge of psychological therapy for mental health difficulties	2.71 (0.87)	2.44 (1.05)
Work with caregivers and other important people	3.31 (0.85)	3.00 (0.85)
End psychological therapy in an effective manner	2.70 (0.85)	2.56 (1.05)

Note.*TCS-ASD/ID* Therapist Confidence Scale. Responses were on a 5-point Likert scale with 0=not at all confident and 4=highly confident. Only clinicians indicating relevant experience were routed to the items.

M, mean; SD, SD deviation.

Among clinicians (n=63) with experience across all three CYP populations (CYP with no autism or intellectual disabilities diagnoses, autistic CYP and CYP with intellectual disabilities), a Friedman test was used to compare self-reported confidence levels when delivering psychological therapy across the three populations. Clinicians rated their confidence about offering effective psychological therapy to CYP with no autism or intellectual disabilities diagnoses (M=2.71, SD=0.92; Mdn=3, IQR=1), autistic CYP (M=2.22, SD=0.81; Mdn=2, IQR=1) and CYP with intellectual disabilities (M=1.81, SD=0.98; Mdn=2, IQR=1). Using only the data from clinicians with experience with all three CYP populations, a Friedman test revealed a significant difference in self-perceived confidence about offering effective psychological therapy to CYP with no autism or intellectual disabilities diagnoses, autistic CYP and CYP with intellectual disabilities (χ^2^(2)=35.822, p<0.001; Kendall’s W=0.284). Post hoc comparison tests using the Wilcoxon signed-rank test indicated clinicians felt statistically significantly less confident about offering effective psychological therapy to CYP with intellectual disabilities than to CYP with no autism or intellectual disabilities diagnoses (p<0.001, r=0.59) and autistic CYP (p=0.038, r=0.31). There was no statistically significant difference in confidence level about offering effective psychological therapy to CYP with no autism or intellectual disabilities diagnoses and autistic CYP (p=0.078, r=0.28).

### Skills

Question four*: do clinicians feel that they have the skills to deliver psychological therapy to individuals with no autism or intellectual disabilities diagnoses, autistic individuals and individuals with intellectual disabilities?*

Among clinicians (n=62) with experience across all three CYP populations (CYP with no autism or intellectual disability diagnoses, autistic CYP and CYP with intellectual disabilities), clinicians rated their skills in delivering psychological therapy differently across the three populations. Clinicians rated their skills about delivering psychological therapy to CYP with no autism or intellectual disabilities diagnoses (M=3.18, SD=0.82; Mdn=3, IQR=1), autistic CYP (M=2.92, SD=0.73; Mdn=3, IQR=0) and CYP with intellectual disabilities (M=2.35, SD=1.07; Mdn=3, IQR=1.25). Using only the data from clinicians with experience with all three CYP populations, a Friedman test revealed a significant difference in self-perceived skills about delivering psychological therapy to CYP with no autism or intellectual disabilities diagnoses, autistic CYP and CYP with intellectual disabilities (χ^2^(2)=34.188, p<0.001; Kendall’s W=0.276). Post hoc comparison tests using Wilcoxon signed-rank test indicated clinicians felt statistically significantly less skilled about delivering psychological therapy to CYP with intellectual disabilities than to CYP with no autism or intellectual disabilities diagnoses (p<0.001, r=0.58) and autistic CYP (p=0.014, r=0.36). There was no statistically significant difference in skills about delivering psychological therapy to CYP with no autism or intellectual disabilities diagnoses and autistic CYP (p=0.240, r=0.22).

### Treatment approaches and perceived evidence base

Question five*: which* psychological therapy *do clinicians believe to be evidence-based for supporting autistic individuals and individuals with intellectual disabilities?*

The following results are based on clinicians with experience delivering psychological therapy to both autistic CYP and CYP with intellectual disabilities (psychological therapy use: autistic CYP n=64, CYP with intellectual disabilities n=61; perceived evidence-based: autistic CYP n=62, CYP with intellectual disabilities n=53). As shown in table 6, the most common psychological therapy used was cognitive behavioural therapy (autism: 54.7%, intellectual disabilities: 47.5%). Clinicians also reported cognitive behavioural therapy to be the most evidence-based (autism: 62.5%, intellectual disabilities: 50.9%). Several additional psychological therapies were endorsed by only a small minority of clinicians and are therefore not presented in table 6.

**Table 6:** Clinician self-reported use and perception of evidence-based psychological therapy for autistic children and young people, and children and young people with intellectual disabilities

Psychological therapy	Usage with autistic children and young people n=64 (%)	Usage for children and young people with intellectual disabilities usage n=61 (%)	Perceived evidence base for autistic children and young people n=62 (%)	Perceived evidence base for children and young people with intellectual disabilities n=53 (%)
Cognitive behavioural therapy	35 (54.7%)	29 (47.5%)	40 (62.5%)	27 (50.9%)
Solution-focused therapy	13 (20.3%)	5 (8.2%)	3 (4.7%)	5 (9.4%)
Other	4 (6.3%)	6 (9.8%)	3 (4.7%)	5 (9.4%)
Talking therapy	2 (3.1%)	6 (9.8%)	1 (1.6%)	2 (3.8%)
Family therapy	0	2 (3.3%)	2 (3.1%)	5 (9.4%)
Dialectical behaviour therapy	1 (1.6%)	0	1 (1.6%)	1 (1.6%)
Narrative therapy	2 (3.1%)	2 (3.3%)	1 (1.6%)	1 (1.6%)
Play therapy	1 (1.6%)	4 (6.6%)	1 (1.6%)	1 (1.6%)

Note. Only clinicians indicating relevant experience were routed to this item. Psychological therapies endorsed by fewer than five respondents across all items were omitted from the table for brevity. These included Lego therpay, sensory moto psychotherapy, emotion-focused therapy, acceptance and commitment therapy, systemic psychotherapy, mindfulness-based cognitive therapy, outdoor therapy, exposure and response prevention and cognitive processing therapy.

### Access criteria

Question six*: what do clinicians report about barriers to access for autistic individuals and individuals with intellectual disabilities?*

Of 135 clinicians overall, clinician self-report of the existence of criteria to accessing mainstream CAMHS questions revealed that 59.3% (n*=*135) of clinicians were responsible for triaging, 37.3% (n=133) for allocating referrals, 42.2% (n=133) for pathway planning and 25.9% (n=135) for making decisions about training and recruitment.

Of 135 clinicians, 20% reported that there were established criteria (eg, exclusionary criteria, practices and policies) for delivering psychological therapy in mainstream CAMHS to autistic CYP and 38.4% for CYP with intellectual disabilities, while nearly half were not sure (autism (n=117): 44.4%, intellectual disabilities (n=64): 34.1%).

## Discussion

Clinicians with experience across the relevant CYP populations reported lower knowledge, skills and confidence when delivering psychological therapy to CYP with intellectual disabilities, compared with autistic CYP and CYP with no autism or intellectual disabilities diagnoses. In addition, clinicians with experience across the relevant CYP populations reported fewer skills to provide psychological therapy to CYP with intellectual disabilities and noted receiving more training for working with autistic CYP than for CYP with intellectual disabilities.

A key implication is that workforce needs may differ by CYP population. Clinicians with experience across the relevant CYP populations reported greater preparedness for working with autistic CYP than for CYP with intellectual disabilities, suggesting that training and supervision may need to be tailored.

Findings among self-reports from clinicians with experience across the relevant CYP populations broadly align with prior international research indicating variability in clinician knowledge, confidence and training when delivering psychological therapy for autism and ADHD populations.^16^ Direct comparison with the Canadian study is limited due to differences in methodology, populations studied and healthcare context.

Although several group differences were statistically significant, statistical significance does not necessarily indicate clinical importance; rather, these findings reflect relative differences in clinician self-reported preparedness across the two CYP populations (autistic CYP and CYP with intellectual disabilities), with mean ratings generally in the moderate range.^35^

### Training

Among clinicians who responded to the training question on whether they had received training to deliver psychological therapy for autistic CYP or CYP with intellectual disabilities, they were more likely to report considering psychological therapy for autistic CYP than for CYP with intellectual disabilities. One possible explanation is the discrepancy in reported training, as over 80% of clinicians reported receiving training to deliver psychological therapy to autistic CYP compared with only 46.9% receiving training for CYP with intellectual disabilities.

This pattern may reflect broader workforce training trends, where autism-focused training appears more widely available within mainstream CAMHS, whereas training related to intellectual disabilities may be less consistently available in these settings.^17^

### Treatment adaptations

Clinicians with relevant experience, across the two CYP populations (autistic CYP and CYP with intellectual disabilities), reported making abstract concepts more concrete as a commonly used adaptation for both populations, reinforcing the benefit of this adapt.^22 36^ Similarly, another common adaptation used was providing structure and predictability, indicating the importance of concrete and structured adjustments. Conversely, adapting treatments to capitalise on strengths and minimise sensory distractions was not commonly used, despite the challenges both groups experience with sensory sensitivities.^11^ This aligns closely with the findings by Poku *et al*,^20^ highlighting the importance of concrete language, visual supports and structured delivery when adapting psychological therapy for CYP with intellectual disabilities.^20^ The least used adaptations were allowing for shorter sessions and involving family members, a valuable resource that is noticeably underutilised,^37^ further highlighting gaps in mental health service delivery models.^18^ The findings highlight the need for more personalised and strength-based approaches.

### Knowledge, confidence, skills

Results from clinicians reporting across the two CYP populations (autistic CYP and CYP with intellectual disabilities) highlighted that clinicians’ reported strengths in empathy and listening, skills vital for developing a therapeutic relationship; however, specialised skills did not score as highly on self-reports, such as using initial mental health treatment assessments and explaining results, which appear to be areas for improvement.

Among clinicians who reported delivering psychological therapy across the three CYP populations (autistic CYP, CYP with intellectual disabilities and CYP with no autism or intellectual disabilities diagnoses), when responding to the knowledge, confidence and skills survey questions, they indicated feeling less knowledgeable and having lower confidence levels, and being less skilled when supporting autistic CYP and CYP with intellectual disabilities compared with supporting autistic CYP and CYP without these diagnoses. Additional training, support and supervision are critical, given that clinicians’ lower confidence levels can affect efficient and equitable service delivery,^16^ especially for CYP with intellectual disabilities.

### Treatment approaches and perceived evidence base

Clinicians with relevant experience reporting across the two CYP populations (autistic CYP and CYP with intellectual disabilities) reported that autistic CYP and CYP with intellectual disabilities are likely to be offered cognitive behavioural therapy as a treatment for mental health conditions, as it is perceived to be both the most used and evidence-based psychological therapy.^19^ However, apparent differences emerged in the perceived evidence base of other psychological therapy for both populations. While creative therapies had lower usage and perceived evidence-based ratings than more traditional approaches (eg, cognitive behavioural therapy), creative therapies still had a higher usage and perceived evidence base than some alternative psychological therapies. However, among clinicians with relevant experience, reporting across the two CYP populations (autistic CYP and CYP with intellectual disabilities), there was a disparity between the perceived evidence base and reported usage rates of creative therapies, which may be due to clinicians’ beliefs about the effectiveness of creative therapies and their clinical practice,^25^ influenced by skills, training and professional backgrounds.

### Access criteria

Previous research has suggested that CYP with intellectual disabilities were more likely to be excluded from mainstream CAMHS. Consistent with clinicians’ survey reports on delivering psychological therapy to autistic CYP and CYP with intellectual disabilities, a higher proportion of respondents identified access criteria for CYP with intellectual disabilities (38.4%) than for autistic CYP (20%).

It may be that CYP with intellectual disabilities are being filtered into other clinical service pathways (eg, intellectual disabilities or neurodevelopmental pathways) rather than through mainstream CAMHS.^38^

The percentage of clinicians who reported uncertainty around access criteria (autism: 44.4%, intellectual disabilities: 34.1%) may suggest that there is a need for clearer communication to clinicians of pathways and thresholds in mainstream CAMHS.^39^

### Limitations

Although these findings provide valuable insight into clinicians’ knowledge, confidence and psychological therapy choices, they also have limitations. While self-report is a widely used method,^32 40^ it is important to consider the potential bias and limitations in this methodology.

Qualitative research, such as interviews,^41^ may provide a detailed understanding of clinicians’ beliefs. The survey did not consider cooccurring diagnoses between these populations, including autism, intellectual disabilities^26^ and other neurodevelopmental conditions (eg, ADHD, dyslexia, dyspraxia, Tourette’s). This oversight may lead to ambiguity in the responses for individuals who fall into both classifications.

As recruitment was coordinated through participating NHS Trusts, the total number of clinicians invited to participate was not available. Therefore, an overall response rate could not be calculated, which may limit the assessment of the sample’s representativeness.

Participating clinicians were predominantly white, female and cognitive behavioural therapists, limiting generalisability. While the professional and demographic characteristics of the current sample were comparable to similar investigations,^16 42^ future research should include more diverse professional groups, geographical areas and local authorities where some mental health support teams are based. Furthermore, it is also important to acknowledge that the present study did not represent all psychological therapies used in mainstream CAMHS, such as creative therapies (eg, art, music, drama, dance and sensory therapy).

## Conclusion

This study found lower self-reported knowledge, confidence and skills among clinicians delivering psychological therapy to CYP with intellectual disabilities compared with autistic CYP and CYP without autism or intellectual disabilities diagnoses. These findings suggest that additional training and support for clinicians working with CYP with intellectual disabilities may warrant consideration. The current study did not directly assess treatment outcomes, service engagement or unmet need, and future research should examine these areas. Reviewing access criteria and ensuring that policies and procedures are clearly communicated to clinicians may help reduce uncertainty regarding access pathways within mainstream CAMHS.

## Supplementary material

10.1136/bmjopen-2026-122190 online supplemental file 1

## Data Availability

Data are available upon reasonable request.
