# Supplementary material for: Psychological therapy for children and young people with a diagnosis of autism or intellectual disabilities in mainstream child and adolescent mental health services: a cross-sectional survey of clinicians’ perspectives in England
Source: BMJ Open. 2026 Nov 2;16(11):e122190. doi: 10.1136/bmjopen-2026-122190 (PMC13735254; doi:10.1136/bmjopen-2026-122190)
Supplement: Supplementary file 1 [file bmjopen-16-11-s001.pdf]

Mental Health Provider Survey

Version 5
14th August 2023
IRAS Number 324236
Sponsor reference number SOC.17/22-23

Clinician Knowledge, Confidence, and Approaches Used in the Provision of Psychological Therapy to Autistic Individuals and Individuals with Intellectual Disability in Child and Adolescent Mental Health Services: Mental Health Provider Survey

Introduction: Thank you for sharing your opinions and experiences with us. This survey will help inform future training opportunities, identify gaps in services, and assess access to mental health services for children and young people with mental health difficulties who also present as Autistic or have an Intellectual Disability.

Participant Information Sheet: https://livewarwickac-my.sharepoint.com/:w:/g/personal/u5500975_live_warwick_ac_uk/EZl2Dnbw2n9HlxYLSRliKbkBLNbw6t1K7VZ1bKoZR8GFow?e=ZGuIB1

* Required

1. **CONSENT FORM**

   Study Name: Clinician Knowledge, Confidence, and Approaches Used in the Provision of Psychological Therapy to Autistic Individuals and Individuals with Intellectual Disability in Child and Adolescent Mental Health Services: Mental Health Provider Survey

   Name of Researcher: Naomi Williams
   Version Number: 2
   Date: 14/08/2023

   Please confirm your full name

2. I confirm that I have read and understand the information sheet (*version 2, 14/08/23*) for the above study. I have had the opportunity to consider the information, ask questions and have had these answered satisfactorily. *

   ◯ Yes

   ◯ No

3. I understand that my participation is voluntary and that I am free to withdraw at any time without giving any reason, without my *medical, social care, or legal rights** being affected. *

   ◯ Yes

   ◯ No

4. I understand that data collected during the study, may be looked at by individuals from The University of Warwick, from regulatory authorities, where it is relevant to my taking part in this study. I give permission for these individuals to have access to my data. *

   ◯ Yes

   ◯ No

5. I am happy for my anonymised data to be used in future research. *

   ◯ Yes

   ◯ No

6. I agree to take part in the above study. *

   ◯ Yes

   ◯ No

7. Full name *

8. Signature

9. Date signed

Start of Block: Information

Thank you for agreeing to participate in our research and helping us learn more about the important work you do with children and young people. There are no right or wrong answers, we want to learn your honest opinion because we understand that everyone works with a unique set of people. Your individual responses will be completely confidential and will not be seen by anyone that you work with.
PLEASE NOTE:

The National Institute for Health and Care Excellence (NICE) provides national guidance and advice for mental health providers.

For the purposes of this study, a **Mental Health Difficulty** refers to difficulties or disorders that affect an individual's mood, thinking, and emotion regulation, and are associated with distress or an impairment in their functioning. As examples, we are referring to any symptoms or diagnoses of anxiety disorder, bipolar disorder, oppositionality, conduct disorders, depression, eating disorders, gender dysphoria, obsessive-compulsive and related disorders, psychosis, post-traumatic stress disorders, panic or substance use. Individuals do not need to have a diagnosed disorder to have mental health difficulties (e.g.an incredibly low mood that causes distress but not all of the symptoms of clinical depression are present). These difficulties or disorders may be mild or severe and may result in an escalation or change in an individual's usual behaviour.
When referring to Mental Health Difficulties, we are NOT referring to conditions such as Autism, or Intellectual Disability.

For the purposes of this study, **Psychological Therapy** refers to a range of interventions, based on psychological concepts and theories, which are designed to help people understand and make changes to their thinking, behaviour and relationships in order to relieve distress and to improve functioning.

This Mental Health Provider Survey relates to **Autistic** Children and Young People (CYP) or Children and Young People (CYP) with an **Intellectual Disability** (ID), also known as Learning Disability, aged **5-18 years old**.
At the end of the survey, you will be given the option to be entered into a prize draw to win either £100 or £50.

End of Block: Information

Start of Block: Demographics

10. **How old are you in years?**

11. **Ethnic Background** *

- Arab.
- Asian or Asian British – Indian.
- Asian or Asian British – Pakistani.
- Asian or Asian British – Bangladeshi.
- Other Asian background.
- Black or Black British – African.
- Black or Black British – Caribbean.
- Other Black background.
- Chinese.
- Gypsy or Traveller.
- Mixed – White and Asian.
- Mixed – White and Black African.
- Mixed – White and Black Caribbean.
- Other mixed background.
- White.
- Other ethnic background.
- Not known.
- Prefer not to say.

12. **Gender** *

- Male
- Female
- Non-binary
- Other
- Prefer not to say

13. Which NHS Regional Team in England do you work in? *

- East of England
- London
- Midlands
- North East and Yorkshire
- North West
- South East
- Other

14. Which NHS Site/Trust in England do you work in? *

- ◯ Nottinghamshire NHS Trust
- ◯ Herefordshire and Worcestershire Health and Care NHS Trust
- ◯ Coventry and Warwickshire Partnership NHS Trust
- ◯ Leicestershire Partnership NHS Trust
- ◯ Surrey Trust
- ◯ South London and Maudsley NHS Foundation Trust
- ◯ North Staffordshire Combined Healthcare NHS Trust
- ◯ Rotherham Doncaster & South Humber (RDaSH) NHS Foundation Trust
- ◯ Chesterfield Royal Hospital NHS Foundation Trust (RFS)
- ◯ The Black Country Healthcare NHS Foundation Trust
- ◯ Hertfordshire Partnership University NHS Foundation Trust
- ◯ Lincolnshire Partnership NHS Foundation Trust
- ◯ Humber Teaching NHS Foundation Trust
- ◯ Northamptonshire Healthcare NHS Foundation Trust
- ◯ Norfolk & Suffolk NHS Foundation Trust
- ◯ Derbyshire Healthcare NHS Foundation Trust
- ◯ Midlands Partnership University NHS Foundation Trust
- ◯ Essex Partnership University Trust
- ◯ Sheffield Children’s NHS Foundation Trust
- ◯ Sheffield Health and Social Care NHS Foundation Trust
- ◯ Cambridgeshire and Peterborough NHS Foundation Trust
- ◯ Sussex Partnership NHS Foundation Trust
- ◯ Birmingham Women's and Children's NHS Foundation Trust
- ◯ Bradford District Care NHS Trust
- ◯ Other

15. If you stated other for question 14, please indicate the name of the trust or site that you work at.

16. Where did you hear about the study? *

- ◯ NIHR CRN Portfolio
- ◯ Employer
- ◯ Word of mouth
- ◯ Social media
- ◯ Other

17. Which service area do you work in? *

- ◯ Mental Health Support Team
- ◯ Intellectual Disability Team
- ◯ Community CAMHS
- ◯ Creative Therapies
- ◯ Crisis
- ◯ Eating Disorder
- ◯ Developmental Neuropsychiatry and Tourette's
- ◯ Other

18. Which Professional Registration or Membership do you hold? *

- ◯ Social Work England
- ◯ Health Care Professions Council
- ◯ Society for Education
- ◯ United Kingdom Council for Psychotherapy
- ◯ British Association for Counselling and Psychotherapy
- ◯ British Association for Behaviour and Cognitive Psychotherapy
- ◯ None
- ◯ Other

19. Which professional group do you belong in? *

- ◯ Child and youth worker (CYW)
- ◯ Registered nurse
- ◯ Recreational therapist
- ◯ Occupational therapist
- ◯ Social worker
- ◯ Clinical psychologist
- ◯ Psychiatrist
- ◯ Counsellor
- ◯ CBT Therapist
- ◯ Education Mental Health Practitioner
- ◯ Wellbeing Practitioner
- ◯ Other

20. How many years of professional practice in your field do you have? *

- ◯ 0-2 years
- ◯ 3-5 years
- ◯ 6-9 years
- ◯ 10-14 years
- ◯ 15-19 years
- ◯ 20+ years

21. What is your primary role? *

- ◯ Direct service practitioner
- ◯ Supervisor/Coordinator
- ◯ Direct-service practitioner and supervisor
- ◯ No direct-service or supervision
- ◯ Other

End of Block: Demographics

Start of Block: Definitions reminder

Definitions

For the next part, you may refer to a **Mental Health Difficulty** as difficulties or disorders that affect an individual's mood, thinking, and emotion regulation, and are associated with distress or an impairment in their functioning and **Psychological Therapy** refers to a range of interventions, based on psychological concepts and theories, which are designed to help people understand and make changes to their thinking, behaviour and relationships in order to relieve distress and to improve functioning.

End of Block: Definitions Reminder

Start of Block: General - Contact

22. How many children/young people do you **CURRENTLY** have on your caseload? *

End of Block: General - Contact

Start of Block: General - Confidence, skills and knowledge

23. How **confident** are you that you can offer effective psychological therapy to children/young people with mental health difficulties, who are **not Autistic** or who **do not** have an **Intellectual Disability**? *

◯ Not at all confident

◯ Slightly confident

◯ Moderately confident

◯ Very confident

◯ Extremely confident

24. I have the **skills** needed to deliver psychological therapy to children/young people with mental health difficulties, who are **not Autistic** or who **do not** have an **Intellectual Disability**. *

◯ Strongly disagree

◯ Disagree

◯ Neither disagree or agree

◯ Agree

◯ Strongly agree

25. Please rate how **knowledgeable** you feel about delivering psychological therapy to children/young people with mental health difficulties, who are **not Autistic** or who **do not** have an **Intellectual Disability**. *

◯ Not at all knowledgeable

◯ Slightly knowledgeable

◯ Moderately knowledgeable

◯ Very knowledgeable

◯ Extremely knowledgeable

End of Block: General - Confidence, skills and knowledge

Start of Block: Autistic children/young people- starting psychological therapy

26. Have you ever provided psychological therapy for **Autistic children/young people or children/young people with Intellectual Disability** (also known as Learning Disability)? *

◯ Yes

◯ No

27. For the next set of questions, think about **Autistic children/young people with mental health difficulties,** that you have worked with in the past or are currently working with.

*

◯ I **have** worked with an Autistic children/young people with mental health difficulties.

◯ I have **never** worked with an Autistic children/young people with mental health difficulties.

28. Approximately what percent of your caseload is spent delivering psychological therapy to **Autistic children/young people** with mental health difficulties? *

◯ 0%

◯ 25%

◯ 50%

◯ 75%

◯ 100%

29. When delivering psychological therapy to **Autistic children/young people,** it would be *(For words below, select strongly disagree, disagree, agree, strongly agree that reflects your opinion.)* *

	Strongly Disagree	Disagree	Agree	Strongly Agı
Good	◯	◯	◯	◯
Effective	◯	◯	◯	◯
Pleasant	◯	◯	◯	◯
Hopeful	◯	◯	◯	◯
Manageable	◯	◯	◯	◯
Bad	◯	◯	◯	◯
Useless	◯	◯	◯	◯
Unpleasant	◯	◯	◯	◯
Hopeless	◯	◯	◯	◯
Burdensome	◯	◯	◯	◯

30. How **likely** would you be to deliver psychological therapy to **Autistic children/young people** with mental health difficulties? *

◯ Extremely unlikely

◯ Quite unlikely

◯ Slightly unlikely

◯ Neither (or not sure)

◯ Slightly likely

◯ Quite likely

◯ Extremely likely

End of Block: Autistic children/young people - starting psychological therapy

Start of Block: Autistic children/young people - Knowledge and confidence

31. How **sure are you** that the psychological therapy chosen for mental health difficulties, being delivered to **Autistic children/young people** is appropriate? *A lot of different things can get in the way of delivering psychological therapy to Autistic children/young people (e.g., the children/young people's or parent's fit with the psychological therapy, scheduling, level of complexity around need or changing circumstances).* *

- Very sure it would not
- Sure it would not
- Uncertain
- Sure it would
- Very sure it would

32. How **confident** are you that you can deliver effective psychological therapy to **Autistic children/young people**? *

- Not at all confident
- Slightly confident
- Moderately confident
- Very confident
- Extremely confident

33. I have the **skills** needed to deliver psychological therapy to **Autistic children/young people** who have mental health difficulties**.** *

- Strongly disagree
- Disagree
- Neither disagree nor agree
- Agree
- Strongly Agree

34. Please rate how **knowledgeable** you feel about delivering psychological therapy to **Autistic children/young people** with mental health difficulties**.** *

- ◯ Not at all knowledgeable
- ◯ Slightly knowledgeable
- ◯ Moderately knowledgeable
- ◯ Very knowledgeable
- ◯ Extremely knowledgeable

35. Please rate how **knowledgeable** you feel about the following topics related to **Autistic children/young people** with mental health difficulties. *

	Not at all knowledgeable	Slightly knowledgeable	Moderately knowledgeable	Very knowledgeable	Extrem knowledg
Core symptoms of Autism	◯	◯	◯	◯	◯
Co-occurring conditions with Autism	◯	◯	◯	◯	◯
Impact of Autistic traits on mental health difficulties	◯	◯	◯	◯	◯
Developing a psychological therapy plan for an Autistic children/young person	◯	◯	◯	◯	◯
Delivering psychological therapy to an Autistic children/young person	◯	◯	◯	◯	◯
Identifying progress toward psychological therapy goals with an Autistic children/young person	◯	◯	◯	◯	◯

36. Within the context of psychological therapies for mental health difficulties, how **confident** are you that you can... *

	Not confident	Slightly confident	Moderately confident	Confident	Highly con
Listen carefully to concerns presented by an Autistic children/young person?	◯	◯	◯	◯	◯
Be empathetic towards an Autistic children/young person?	◯	◯	◯	◯	◯
Understand special issues related to having Autism and their impact on a children/young person's life?	◯	◯	◯	◯	◯
Communicate with an Autistic children/young person?	◯	◯	◯	◯	◯
Develop a therapeutic relationship with an Autistic children/young person?	◯	◯	◯	◯	◯
Gather information from an Autistic children/young person, so that their difficulties can be better understood?	◯	◯	◯	◯	◯
Use assessments in a way that an Autistic children/young person will understand?	◯	◯	◯	◯	◯
Explain results of an assessment process to an Autistic children/young person?	◯	◯	◯	◯	◯
Use knowledge about mental health difficulties in formulating the needs of an Autistic children/young person?	◯	◯	◯	◯	◯
Help an Autistic children/young person to identify issues that need to be considered in sessions?	◯	◯	◯	◯	◯
Use knowledge of psychological					

of psychological therapies for mental health difficulties, to work effectively with an Autistic children/young person?	◯	◯	◯	◯	◯
Identify psychological therapies that will be effective for an Autistic children/young person?	◯	◯	◯	◯	◯
Work with caregivers and other important people in the lives of Autistic children/young person?	◯	◯	◯	◯	◯
End psychological therapy with an Autistic children/young person in an effective manner?	◯	◯	◯	◯	◯

End of Block: Autistic children/young people - Knowledge and confidence

Start of Block: Autistic children/young people - Experience

37. In your experience of working with **Autistic children/young people** with mental health difficulties, what are their most common presenting conditions? Please select up to 3 *

Please select at most 3 options.

- ☐ Anxiety
- ☐ Low mood
- ☐ Challenging behaviours (e.g., aggression, irritability)
- ☐ Depression
- ☐ Eating disorders
- ☐ Gender dysphoria
- ☐ Obsessive-compulsive and related disorders
- ☐ Psychosis
- ☐ PTSD
- ☐ Substance use
- ☐ Other

38. When working with **Autistic children/young people** with mental health difficulties, which of the following psychological therapies have you **used**? Please select all that apply. *

- ☐ Solution focused therapy
- ☐ Cognitive behavioural therapy
- ☐ Emotion focused therapy
- ☐ Narrative therapy
- ☐ Talking therapy
- ☐ Acceptance and commitment therapy
- ☐ Family therapy
- ☐ Dialectical behaviour therapy
- ☐ Dyadic developmental psychotherapy
- ☐ Parent child interaction therapy
- ☐ Exposure and response prevention
- ☐ Prolonged exposure therapy
- ☐ Sensory motor psychotherapy
- ☐ Cognitive processing therapy
- ☐ Progressive counting
- ☐ Play therapy
- ☐ Lego therapy
- ☐ Mindfulness Based Cognitive Therapy
- ☐ Systemic psychotherapy
- ☐ Outdoor therapy
- ☐ Other

39. Please select which of the approaches are **evidenced based** for delivering psychological therapy to **Autistic children/young people** with mental health difficulties? *

- [ ] Solution focused therapy
- [ ] Cognitive behavioural therapy
- [ ] Emotion focused therapy
- [ ] Narrative therapy
- [ ] Talking therapy
- [ ] Acceptance and commitment therapy
- [ ] Family therapy
- [ ] Dialectical behaviour therapy
- [ ] Dyadic developmental psychotherapy
- [ ] Parent child interaction therapy
- [ ] Exposure and response prevention
- [ ] Prolonged exposure therapy
- [ ] Sensory motor psychotherapy
- [ ] Cognitive processing therapy
- [ ] Progressive counting
- [ ] Play therapy
- [ ] Lego therapy
- [ ] Mindfulness Based Cognitive Therapy
- [ ] Systemic psychotherapy
- [ ] Outdoor therapy
- [ ] Other

40. Of the **Autistic children/young people** with mental health difficulties on your typical caseload, what percent of them have complex mental health conditions? (Complex Mental Health Conditions are defined as conditions which are very severe, there are significant challenges with treating them, and several other factors often further complicate their illnesses.)
*

	0%	20%	40%	60%	80%	10(
None of them	○	○	○	○	○	○
About half of them	○	○	○	○	○	○
All of them	○	○	○	○	○	○

End of Block: Autistic children/young people – Experience

Start of Block: Autistic children/young people - Past Training

41. Have you received any training for delivering psychological therapy to **Autistic children/young people** (e.g., CPD workshop, in-service training, academic)? *

- Yes
- No

End of Block: Autistic children/young people - Past Training

Start of Block: Autistic children/young people - Adaptions

42. In the past, what **adaptations** have you made when delivering psychological therapy with **Autistic children/young people** with mental health difficulties? (select all that apply) *

- ☐ Make abstract concepts more concrete
- ☐ Provide detailed information
- ☐ Provide structure and predictability (e.g., routines, transition activities)
- ☐ Support with processing verbal information (e.g., simplifying language)
- ☐ Capitalise on strengths (e.g., intelligence and acquisition of new information)
- ☐ Make use of special interests (e.g., include individual interests as part of therapy)
- ☐ Provide opportunities for generalisation and ongoing practice
- ☐ Minimise sensory distraction (e.g., adjusting lights and sounds)
- ☐ Minimise executive functioning difficulties
- ☐ Reduce anxiety with distancing techniques
- ☐ Length of sessions: Shorter
- ☐ Length of sessions: Longer
- ☐ Environment (e.g., sensory area, relaxation area: mats or bean bag chair, visual aids, use of outdoor space)
- ☐ Involving family members in sessions
- ☐ Developing awareness of Interoception (The sense of our internal state such as, organs and feeling and emotions.) and Proprioception (The sense of our body in the space around us.)
- ☐ None
- ☐ Other

End of Block: Autistic children/young people - Adaptions

Start of Block: children/young people with Intellectual Disability - starting psychological therapy

43. For the next set of questions, the same questions that were asked for Autistic CYP will now be asked for working with **children/young people with an Intellectual Disability (ID), also known as Learning Disability, who also present with mental health difficulties.** *

- ◯ I **have** worked with **children/young people with an Intellectual Disability**.
- ◯ I have **never** worked with **children/young people with an Intellectual Disability.**

44. Approximately what percent of your caseload is spent delivering psychological therapy to **children/young people with an Intellectual Disability** with mental health difficulties? *

- ◯ 0%
- ◯ 25%
- ◯ 50%
- ◯ 75%
- ◯ 100%

45. When delivering psychological therapy to **children/young people with an Intellectual Disability**, it would be *(For words below, select strongly disagree, disagree, agree, strongly agree that reflects your opinion.)* *

	Strongly Disagree	Disagree	Agree	Strongly Agı
Good	◯	◯	◯	◯
Effective	◯	◯	◯	◯
Pleasant	◯	◯	◯	◯
Hopeful	◯	◯	◯	◯
Manageable	◯	◯	◯	◯
Bad	◯	◯	◯	◯
Useless	◯	◯	◯	◯
Unpleasant	◯	◯	◯	◯
Hopeless	◯	◯	◯	◯
Burdensome	◯	◯	◯	◯

46. How **likely** would you be to deliver psychological therapy to **children/young people with an Intellectual Disability** with mental health difficulties? *

- Extremely unlikely
- Quite unlikely
- Slightly unlikely
- Neither (or not sure)
- Slightly likely
- Quite likely
- Extremely likely

End of Block: children/young people with an ID- starting psychological therapy

Start of Block: children/young people with an Intellectual Disability - Knowledge and confidence

47. How **sure are you** that the psychological therapy chosen for mental health difficulties, being delivered to **children/young people with an Intellectual Disability** is appropriate? *A lot of different things can get in the way of delivering psychological therapy to children/young people with an Intellectual Disability (e.g., the children/young people's or parent's fit with the psychological therapy, scheduling, level of complexity around need or changing circumstances).*
*

- Very sure it would not
- Sure it would not
- Uncertain
- Sure it would
- Very sure it would

48. How **confident** are you that you can deliver effective psychological therapy to **children/young people with an Intellectual Disability**? *

- Not at all confident
- Slightly confident
- Moderately confident
- Very confident
- Extremely confident

49. I have the **skills** needed to deliver psychological therapy to **children/young people with an Intellectual Disability** who have mental health difficulties**.** *

- Strongly disagree
- Disagree
- Neither disagree nor agree
- Agree
- Strongly Agree

50. Please rate how **knowledgeable** you feel about delivering psychological therapy to **children/young people with an Intellectual Disability** with mental health difficulties**.** *

- ◯ Not at all knowledgeable
- ◯ Slightly knowledgeable
- ◯ Moderately knowledgeable
- ◯ Very knowledgeable
- ◯ Extremely knowledgeable

51. Please rate how **knowledgeable** you feel about the following topics related to **children/young people with an Intellectual Disability** with mental health difficulties. *

	Not at all knowledgeable	Slightly knowledgeable	Moderately knowledgeable	Very knowledgeable	Extrem knowledg
Core symptoms of Intellectual Disability	◯	◯	◯	◯	◯
Co-occurring conditions with Intellectual Disability	◯	◯	◯	◯	◯
Impact of Intellectual Disability traits on mental health difficulties	◯	◯	◯	◯	◯
Developing a psychological therapy plan for children/young people with an Intellectual Disability	◯	◯	◯	◯	◯
Delivering psychological therapy to a children/young person with an Intellectual Disability	◯	◯	◯	◯	◯
Identifying progress toward psychological therapy goals with a children/young person with an Intellectual Disability	◯	◯	◯	◯	◯

52. Within the context of psychological therapies for mental health difficulties, how **confident** are you that you can... *

	Not confident	Slightly confident	Moderately confident	Confident	Highly con
Listen carefully to concerns presented by a children/young person with an Intellectual Disability?	◯	◯	◯	◯	◯
Be empathetic towards a children/young people with an Intellectual Disability?	◯	◯	◯	◯	◯
Understand special issues related to having an Intellectual Disability and their impact on a child/young person's life?	◯	◯	◯	◯	◯
Communicate with a children/young person with an Intellectual Disability?	◯	◯	◯	◯	◯
Develop a therapeutic relationship with a children/young person with an Intellectual Disability?	◯	◯	◯	◯	◯
Gather information from children/young people with an Intellectual Disability, so that their difficulties can be better understood?	◯	◯	◯	◯	◯
Use assessments in a way that a children/young person with an Intellectual Disability will understand?	◯	◯	◯	◯	◯
Explain results of an assessment process to a children/young person with an Intellectual Disability?	◯	◯	◯	◯	◯
Use knowledge about mental health difficulties in formulating the needs of a children/young people with an Intellectual Disability?	◯	◯	◯	◯	◯

Disability?

Help a children/young person with an Intellectual Disability to identify issues that need to be considered in sessions?

Use knowledge of psychological therapies for mental health difficulties, to work effectively with a children/young person with an Intellectual Disability?

Identify psychological therapies that will be effective for a children/young person with an Intellectual Disability?

Work with caregivers and other important people in the lives of children/young people with an Intellectual Disability?

End psychological therapy with a children/young person with an Intellectual Disability in an effective manner?

Er... children/young people with ID - Knowledge and confidence

Sta... n/young people with an Intellectual Disability - Experience

53. ...**ence** of working with **children/young people with an Intellectual Disability** ...alth difficulties, what are their most common presenting conditions? Please

...st 3 options.

...behaviours (e.g., aggression, irritability)

...ders

...phoria

...ompulsive and related disorders

...se

54. When working with **children/young people with an Intellectual Disability** with mental health difficulties, which of the following psychological therapies have you **used**? Please select all that apply. *

- ☐ Solution focused therapy
- ☐ Cognitive behavioural therapy
- ☐ Emotion focused therapy
- ☐ Narrative therapy
- ☐ Talking therapy
- ☐ Acceptance and commitment therapy
- ☐ Family therapy
- ☐ Dialectical behaviour therapy
- ☐ Dyadic developmental psychotherapy
- ☐ Parent child interaction therapy
- ☐ Exposure and response prevention
- ☐ Prolonged exposure therapy
- ☐ Sensory motor psychotherapy
- ☐ Cognitive processing therapy
- ☐ Progressive counting
- ☐ Play therapy
- ☐ Lego therapy
- ☐ Mindfulness Based Cognitive Therapy
- ☐ Systemic psychotherapy
- ☐ Outdoor therapy
- ☐ Other

55. Please select which of the approaches are **evidenced based** for delivering psychological therapy to **children/young people with an Intellectual Disability** with mental health difficulties. *

- ☐ Solution focused therapy
- ☐ Cognitive behavioural therapy
- ☐ Emotion focused therapy
- ☐ Narrative therapy
- ☐ Talking therapy
- ☐ Acceptance and commitment therapy
- ☐ Family therapy
- ☐ Dialectical behaviour therapy
- ☐ Dyadic developmental psychotherapy
- ☐ Parent child interaction therapy
- ☐ Exposure and response prevention
- ☐ Prolonged exposure therapy
- ☐ Sensory motor psychotherapy
- ☐ Cognitive processing therapy
- ☐ Progressive counting
- ☐ Play therapy
- ☐ Lego therapy
- ☐ Mindfulness Based Cognitive Therapy
- ☐ Systemic psychotherapy
- ☐ Outdoor therapy
- ☐ Other

56. Of the **children/young people with an Intellectual Disability** with mental health difficulties on your typical caseload, what percent of them have complex mental health conditions? (Complex Mental Health Conditions are defined as conditions which are very severe, there are significant challenges with treating them, and several other factors often further complicate their illnesses.)
*

	0%	20%	40%	60%	80%	10(
None of them	○	○	○	○	○	○
About half of them	○	○	○	○	○	○
All of them	○	○	○	○	○	○

End of Block: children/young people with an ID – Experience

Start of Block: children/young people with an Intellectual Disability - Past Training

57. Have you received any training for delivering psychological therapy to **children/young people with an Intellectual Disability** (e.g., CPD workshop, in-service training, academic)? *

◯ Yes

◯ No

End of Block: children/young people with an ID - Past Training

Start of Block: children/young people with an Intellectual Disability - Adaptions

58. In the past, what **adaptations** have you made when delivering psychological therapy with **children/young people with an Intellectual Disability** with mental health difficulties? (select all that apply) *

- ☐ Make abstract concepts more concrete
- ☐ Provide detailed information
- ☐ Provide structure and predictability (e.g., routines, transition activities)
- ☐ Support with processing verbal information (e.g., simplifying language)
- ☐ Capitalise on strengths (e.g., intelligence and acquisition of new information)
- ☐ Make use of special interests (e.g., include individual interests as part of therapy)
- ☐ Provide opportunities for generalisation and ongoing practice
- ☐ Minimise sensory distraction (e.g., adjusting lights and sounds)
- ☐ Minimise executive functioning difficulties
- ☐ Reduce anxiety with distancing techniques
- ☐ Length of sessions: Shorter
- ☐ Length of sessions: Longer
- ☐ Environment (e.g., sensory area, relaxation area: mats or bean bag chair, visual aids, use of outdoor space)
- ☐ Involving family members in sessions
- ☐ Developing awareness of Interoception (The sense of our internal state such as, organs and feeling and emotions.) and Proprioception (The sense of our body in the space around us.)
- ☐ None
- ☐ Other

End of Block: children/young people with an ID - Adaptions

Start of Block: Organisation Procedures

59. To your knowledge, does your service have established criteria (e.g., exclusionary criteria, practices, policies) for providing mental health services to **Autistic children/young people?** Participant Information Sheet for your information https://livewarwickac-my.sharepoint.com/:w:/g/personal/u5500975_live_warwick_ac_uk/EUxZiOVY7kBErXZPkf2J6kQBScbIXD9l-2FfEh_v6syMoA?e=pKavMD *

   - Yes (please give details in other)
   - No
   - Not sure
   - Other

60. To your knowledge, does your service have established criteria (e.g., exclusionary criteria, practices, policies) for providing mental health services to **children/young people with an Intellectual Disability?** Participant Information Sheet for your information https://livewarwickac-my.sharepoint.com/:w:/g/personal/u5500975_live_warwick_ac_uk/EUxZiOVY7kBErXZPkf2J6kQBScbIXD9l-2FfEh_v6syMoA?e=pKavMD *

   - Yes (please give details in other)
   - No
   - Not sure
   - Other

End of Block: Organisation Procedures

Start of Block: Triage, allocations and pathways

61. Do you have an active role in **triaging** referrals? *

◯ Yes

◯ No

62. Do you have a role in **allocating** referrals to clinicians? *

◯ Yes

◯ No

63. Do you have a role in **pathway planning** and **care bundles** for your service? *

◯ Yes

◯ No

64. Do you make any **decisions** about **training** and **recruitment** for the service? *

◯ Yes

◯ No

End of Block: Triage, allocations and pathways

Start of Block: Personal experiences

65. Do you know someone **who is Autistic or has an Intellectual Disability, also known as a Learning Disability** outside of your work (i.e., individuals in your **personal** circle such as family, friends, etc.)? *

- Yes
- No

66. On average, how frequent is your contact with **Autistic** individuals **or** individuals with an **Intellectual Disability, also known as a Learning Disability** outside of your work (i.e., individuals in your **personal** circle such as family, friends, etc.)? Please select the most frequent option. *

- Very rarely
- Rarely
- Occasionally
- Frequently
- Very frequently

67. Would you consider yourself to be **Autistic** or have an **Intellectual Disability, also known as a Learning Disability**? *

- Yes
- No
- Prefer not to disclose

68. Have you previously had or currently have a **mental health condition**? *

- Yes
- No
- Prefer not to disclose

End of Block: Personal experiences

Start of Block: Prize draw entry, further research

69. Are you interested in entering the prize draw, or the results of this study, or being contacted for future research? If you answered yes please click link to access entry https://forms.office.com/e/GHMrWT1L0J and remember to submit the Mental Health Provider Survey responses before closing this window.

- ◯ Yes
- ◯ No

End of Block: Prize draw, further research

END

This content is neither created nor endorsed by Microsoft. The data you submit will be sent to the form owner.

Microsoft Forms
