# Supplementary material for: Psychological therapy for children and young people with a diagnosis of autism or intellectual disabilities in mainstream child and adolescent mental health services: a cross-sectional survey of clinicians’ perspectives in England
Source: BMJ Open. 2026 Nov 2;16(11):e122190. doi: 10.1136/bmjopen-2026-122190 (PMC13735254; doi:10.1136/bmjopen-2026-122190)
Supplement: Supplementary file 2 [file bmjopen-2026-122190.reviewer_comments.pdf]

PEER REVIEW HISTORY

BMJ Open publishes all reviews undertaken for accepted manuscripts. Reviewers are asked to complete a checklist review form and are provided with free text boxes to elaborate on their assessment. These free text comments are reproduced below.

ARTICLE DETAILS

Title (Provisional)

Psychological Therapy for Children and Young People with a Diagnosis of Autism or Intellectual Disabilities in Mainstream Child and Adolescent Mental Health Services: A Cross-Sectional Survey of Clinicians' Perspectives in England

Authors

Williams, Naomi; Tuomainen, Helena; Taylor, Julie; Weiss, Jonathan; Gray, Kylie M.; Trower, Hayley L.; Pandher, Parneet; Smith, Kayla; Crawford, Hayley

---

VERSION 1 - REVIEW

**Reviewer**	**1**
**Name**	**Fullwood, Catherine**
**Affiliation**	**Manchester University NHS Foundation Trust, Research and Innovation**
**Date**	**15-Jun-2026**
**COI**	**NA**

BMJ open review 15626

Thank you for the opportunity to review this interesting paper. Overall, I found the content to be of interest and generally well written, however I have some concerns around the appropriateness of some of the methods which may be due to a lack of clarity in the precise objectives, but may also be errors due to non-robust assumptions.

Methods – Recruitment

In the last paragraph please change "its" to "their" in the sentence "..Trust research and development teams who decided on its own process for informing Procedure local CAMHS.."

Online Mental Health Provider Survey

Please rephrase the section at the bottom of page 9 – whilst I can see what you are trying to say, it doesn't flow properly to jump from the example of the built in skip logic to a list of features.

This section may also benefit from using some of the sentences identified later regarding the survey selection of relevant questions based on previous responses.

General Methods

Please add a sentence to the methods to clarify the study design for the reader. An initial line should state. “This is an observation cohort survey study.” You should also make it clear what the full aims and objectives are. I understand the overall aim, but it’s not entirely clear what the exact comparisons are that you want to make and this makes it hard to assess whether your assumptions made in the statistical methods are valid.

There is no section which talks about ethics or any form of approvals for the research.

Whilst I understand the overall research question, the individual objectives and subsequently outcome measures are not clear. This also makes it difficult to understand whether the statistical methods are appropriate to answer the questions.

Statistical methods

Are the two samples truly independent for those clinicians responding regarding their self-reported knowledge for working with autistic CYP or those with intellectual disabilities? From the numbers later there seems to be an overlap in the groups and

whilst I appreciate that these are different patients, I suspect there may be an overlap in how individuals gather knowledge.

Responses were treated as independent observations of clinical experiences across the three populations in which they had relevant experience (e.g., autism, intellectual disabilities, and no autism or intellectual disabilities diagnosis) – Is this a valid assumption and what is your reasoning for making it?

Limitations

You only have 18 individuals responding to those without autism or intellectual disabilities. This is small and noticeable different to the other groups, this will result in weaker comparisons with this group.

Results

Are the response rates different due to missing data or sections which are not relevant for some individuals? Can you provide more information about the rates – for example what is the range of responses? Is data missing at random or not? Does this relate solely to the next paragraph or is there variation in the response rates for other reasons?

Given that there are 135 individuals in total and 117 have experience with autistic CYP and 64 with those with intellectual disabilities, I have some concerns around your assumption of independence between the groups.

Do the 18 overlap either group? Please give percentages and clarify where there is overlap.

“Response scores for items relating to CYP with intellectual disabilities were lower because clinicians without applicable experience were automatically routed past these items.” This should be made clear in the methods and then when you interpret the results you can state this when you talk about the response rates by incorporating it into a sentence.

“Participants responded to the survey items, about their previous and/or current experiences of working in mainstream CAMHS even if they were not currently based in those services.” – Again to me this is more a survey description / methods sentence. Here you just need to clarify response rates and the actual results.

(M_{age} = 39 years, SD = 10.03). M_{age} is not a common abbreviation. Please define, or preferably just state (mean age 39 years, SD 10.03). The standard deviation does not need to be to 2 decimal places given you have reported mean age to 0. Both are calculations.

“with less than half (37.8%)” – I would call this just over one third.

31.1 + 21.5 – this is only just over half, I would not call this most? If 16.3 do not hold a professional membership what about the remainder?

“Item non-response reflected planned skip patterns within the survey design, rather than missing data.” – Providing this information higher would remove y earlier point.

“In total, 117 clinicians responded about receiving training to deliver psychological therapy to autistic CYP and 64 clinicians responded regarding people with intellectual disabilities.” – This is repetitive from above and not needed.

“Clinicians who reported the likelihood of delivering psychological therapy, was lower for CYP…..” Please remove the “.”

Please check these percentages or make it clear what they are out of– “As shown in Table 2, 43% (n= 53) of clinicians reported that they were extremely likely to deliver psychological therapy for autistic CYP; however, 43.7% (n= 48) reported that they were uncertain whether the chosen psychological therapy were appropriate. 5.9% (n= 8) reported that they were extremely likely to deliver psychological therapy for CYP with intellectual disabilities; however, 23.6% (n= 28) were uncertain whether the chosen psychological therapy were appropriate.” – 53/117*100 doesn’t equal 43%? 8/64*100 doesn’t equal 5.9%

Table 2 – This reflects my above comment about percentages. They don’t add up to 100% which I would expect. Please clarify.

Training – what percentage answered yes to both? How many did not answer this question at all – it is important to give the denominators.

I presume in Table 3 they were able to select more than one option?

Knowledge – in terms of interpretation the actual values are more meaningful than the z-score. Either than or reporting a difference. Also you use the mean (SD), but choose a non-parametric test. Especially given the categorical nature of this scale median (IQR) would be more informative. Again here I'm concerned about the overlap between groups. Did you repeat the tests with those with true independence?

You are also doing repeat tests on this domain – this could mean that some of your findings are due to chance. A crude correction would be to divide the p-value by the number of tests, i.e. use the value 0.008 instead. This would mean that only the top two and bottom one would remain truly significant.

What is H(2)?

As before "M" is not a standard abbreviation for mean.

Table 5 – Are these the mean of the scale? What is the distribution like? Often scales are skewed.

I have similar queries to those raised for knowledge for confidence and skills.

"There were no statistically significant differences in clinician-reported levels of confidence in working with different groups of CYP (p = .424)." – This doesn't follow-on from the previous section.

"Therefore, there was no statistically significant difference (p = .049)" – What does this relate to?

Table 6 – Why do you choose to report those percentages for 2, 1 and 0 as <2%. 0 could be 0%, 1 0.9% etc?

Could individuals only select one option? If not, how many selected multiple?

Discussion

"Although several group differences were statistically significant, statistical significance does not necessarily indicate clinical importance; rather, these findings reflect relative differences in clinician self-reported preparedness, with mean ratings generally in the moderate range (Gikandi et al., 2024)."

This is true, you need to ensure you interpret your findings clinically also.

How many individuals report training in both? How does the different uptake reflect availability in different areas? Are there any differences by type of individual / where they are based?

Access criteria

Please revise this sentence. "In line with the current survey suggested that a higher proportion of respondents highlighted access criteria for CYP with intellectual disabilities (38.4%) than.."

Overall, whilst I believe that your conclusions are likely to be correct, I have concerns around some of the assumptions in the statistical methods and some of the presentation. I feel that this needs to be reviewed before concluding. Have you had any conversations with a statistician regarding the most appropriate way to answer your questions and display the findings?

**Reviewer**	**2**
**Name**	**Da-Silva-Ellimah, Morenike**
**Affiliation**	**University of Leicester**
**Date**	**23-Jun-2026**
**COI**	**Yes- For transparency, I note that I am on the same**

Wellcome Trust PhD programme as the author, although we are in different cohorts and based at different universities.

Thank you for the opportunity to review this manuscript. The paper addresses an important knowledge gap in CAMHS research, and the incorporation of PPI meaningfully strengthens the relevance of the work to affected children, young people, and families. Overall, the manuscript is well-constructed, but it would benefit from minor revisions to enhance clarity.

1. Define autism spectrum disorder and intellectual disability to ensure consistency and accessibility for readers who may not be specialists in neurodevelopmental conditions.

2. State the regions represented in the sample and the number of respondents from each region to provide insight into potential geographic variation in clinician perspectives.

3. Specify the clinician types by region and note whether there were differences in professional backgrounds across regions, as this may influence interpretation of the findings.

Thank you again for the invitation to review this insightful and valuable manuscript.

VERSION 1 - AUTHOR RESPONSE

Reviewer 1:

Thank you for the opportunity to review this interesting paper. Overall, I found the content to be of interest and generally well written, however I have some concerns around the appropriateness of some of the methods which may be due to a lack of clarity in the precise objectives, but may also be errors due to non-robust assumptions.

We appreciate your thorough review of our manuscript. We have considered your feedback and believe you will be pleased with the revised version of this paper.

Methods – Recruitment

In the last paragraph please change “its” to “their” in the sentence “..Trust research and development teams who decided on its own process for informing Procedure local CAMHS..”

This has been updated.

Online Mental Health Provider Survey

Please rephrase the section at the bottom of page 9 – whilst I can see what you are trying to say, it doesn’t flow properly to jump from the example of the built in skip logic to a list of features.

This section may also benefit from using some of the sentences identified later regarding the survey selection of relevant questions based on previous responses.

We have rephrased the section and added in further clarity, I have also improved the flow with an orienting sentence to link to the eight domains outlined following this section.

General Methods

Please add a sentence to the methods to clarify the study design for the reader. An initial line should state. “This is an observation cohort survey study.”

You should also make it clear what the full aims and objectives are. I understand the overall aim, but it’s not entirely clear what the exact comparisons are that you want to make and this makes it hard to assess whether your assumptions made in the statistical methods are valid.

There is no section which talks about ethics or any form of approvals for the research.

Whilst I understand the overall research question, the individual objectives and subsequently outcome measures are not clear. This also makes it difficult to understand whether the statistical methods are appropriate to answer the questions.

Thank you for these helpful suggestions. We have revised the Methods section to clarify the study design as an observational cross-sectional survey study. We have also expanded the opening of the Methods to clearly state the study objectives and primary outcome measures, providing a clearer link between the research questions and the statistical analyses. In addition, we have added a dedicated Ethical Approval section to improve clarity regarding the research approvals and participant consent procedures.

Statistical methods

Are the two samples truly independent for those clinicians responding regarding their self-reported knowledge for working with autistic CYP or those with intellectual disabilities? From the numbers later there seems to be an overlap in the groups and whilst I appreciate that these are different patients, I suspect there may be an overlap in how individuals gather knowledge.

Updated and addressed the overlap issue.

Responses were treated as independent observations of clinical experiences across the three populations in which they had relevant experience (e.g., autism, intellectual disabilities, and no autism or intellectual disabilities diagnosis) – Is this a valid assumption and what is your reasoning for making it?

The samples for the clinicians with experiences for the three patient groups (CYP with no autism or intellectual disabilities diagnoses, CYP with autism, and CYP with intellectual disabilities) indeed overlapped. We have changed the analysis methods to Wilcoxon signed rank test and Friedman test.

Limitations

You only have 18 individuals responding to those without autism or intellectual disabilities. This is small and noticeable different to the other groups, this will result in weaker comparisons with this group.

Thank you for this comment. We would like to clarify that the comparison group for children and young people (CYP) without autism or intellectual disabilities comprised all clinicians (n = 135), rather than only the additional 18 clinicians. The additional 18 clinicians contributed responses only to the comparison group because they did not report experience working with autistic CYP or CYP with intellectual disabilities. We have revised the Methods and Results to clarify the composition of each comparison group and how participants contributed responses across the survey.

Results

Are the response rates different due to missing data or sections which are not relevant for some individuals? Can you provide more information about the rates – for example what is the range of responses? Is data missing at random or not? Does this relate solely to the next paragraph or is there variation in the response rates for other reasons?

Thank you for this comment. We have clarified in the Methods that the survey incorporated built-in skip logic, whereby participants were automatically routed to questions relevant to their reported clinical experience. Consequently, differences in response rates primarily reflect planned survey design rather than missing data. We have also clarified this in the Results by stating that lower response rates for certain survey items were due to the planned skip logic rather than participant non-response.

Given that there are 135 individuals in total and 117 have experience with autistic CYP and 64 with those with intellectual disabilities, I have some concerns around your assumption of independence between the groups.

The samples for the clinicians with experiences for the three patient groups (CYP with no autism or intellectual disabilities diagnoses, CYP with autism, and CYP with intellectual disabilities) indeed overlapped. We have changed the analysis methods to Wilcoxon signed rank test and Friedman test.

Do the 18 overlap either group? Please give percentages and clarify where there is overlap.

Thank you for this comment. We have clarified the composition of the comparison groups in the Methods. All participants (n = 135) completed questions relating to children and young people (CYP) without autism or intellectual disabilities. Clinicians with relevant experience additionally completed survey sections relating to autistic CYP (n = 117) and/or CYP with intellectual disabilities (n = 64), according to the survey's built-in skip logic. We have also clarified the overlap between these groups in the manuscript.

"Response scores for items relating to CYP with intellectual disabilities were lower because clinicians without applicable experience were automatically routed past these items." This should be made clear in the methods and then when you interpret the results you can state this when you talk about the response rates by incorporating it into a sentence.

Thank you for this helpful suggestion. We have clarified in the Methods that the survey incorporated built-in skip logic, whereby clinicians were automatically routed to questions relevant to their reported clinical experience. We have also revised the Results pg.15. to clarify that the lower response rates for items relating to CYP with intellectual disabilities reflect the planned survey design, rather than missing data, and have incorporated this explanation into the interpretation of the response rates.

"Participants responded to the survey items, about their previous and/or current experiences of working in mainstream CAMHS even if they were not currently based in those services." – Again to me this is more a survey description / methods sentence. Here you just need to clarify response rates and the actual results.

Thank you for your comment. We have retained this as it helps to further clarify what the results are about, as the reader.

(M_{age} = 39 years, SD = 10.03). M_{age} is not a common abbreviation. Please define, or preferably just state (mean age 39 years, SD 10.03). The standard deviation does not need to be to 2 decimal places given you have reported mean age to 0. Both are calculations.

We have changed it to mean age.

"with less than half (37.8%)" – I would call this just over one third.

We have changed this.

31.1 + 21.5 – this is only just over half, I would not call this most? If 16.3 do not hold a professional membership what about the remainder?

This has been updated and presented in table 1.

"Item non-response reflected planned skip patterns within the survey design, rather than missing data." – Providing this information higher would remove y earlier point.

Updated.

“In total, 117 clinicians responded about receiving training to deliver psychological therapy to autistic CYP and 64 clinicians responded regarding people with intellectual disabilities.” – This is repetitive from above and not needed.

Updated.

“Clinicians who reported the likelihood of delivering psychological therapy, was lower for CYP…..” Please remove the “.”

Removed.

Please check these percentages or make it clear what they are out of– “As shown in Table 2, 43% (n= 53) of clinicians reported that they were extremely likely to deliver psychological therapy for autistic CYP; however, 43.7% (n= 48) reported that they were uncertain whether the chosen psychological therapy were appropriate. 5.9% (n= 8) reported that they were extremely likely to deliver psychological therapy for CYP with intellectual disabilities; however, 23.6% (n= 28) were uncertain whether the chosen psychological therapy were appropriate.” – 53/117*100 doesn’t equal 43%? 8/64*100 doesn’t equal 5.9%

We have corrected the %.

Table 2 – This reflects my above comment about percentages. They don’t add up to 100% which I would expect. Please clarify.

We have corrected the %.

Training – what percentage answered yes to both? How many did not answer this question at all – it is important to give the denominators.

We have added the numbers.

I presume in Table 3 they were able to select more than one option?

Yes, this has been clarified in the manuscript.

Knowledge – in terms of interpretation the actual values are more meaningful than the z-score. Either than or reporting a difference. Also you use the mean (SD), but choose a non-parametric test. Especially given the categorical nature of this scale median (IQR) would be more informative.

While non-parametric tests were required due to non-normal distributions, mean and standard deviation are reported descriptively to maximise data granularity and capture subtle shifts in distribution. Medians would obscure these nuanced differences, whereas the mean and standard deviation reflect the exact weight and full variance of all responses.

Again here I’m concerned about the overlap between groups. Did you repeat the tests with those with true independence?

The samples for the clinicians with experiences for the three patient groups (CYP with no autism or intellectual disabilities diagnoses, CYP with autism, and CYP with intellectual disabilities) indeed overlapped. We have changed the analysis methods to Wilcoxon signed rank test and Friedman test.

You are also doing repeat tests on this domain – this could mean that some of your findings are due to chance. A crude correction would be to divide the p-value by the number of tests, i.e. use the value 0.008 instead. This would mean that only the top two and bottom one would remain truly significant.
Bonferroni correction was employed.

What is H(2)?

As before "M" is not a standard abbreviation for mean.

Table 5 – Are these the mean of the scale? What is the distribution like? Often scales are skewed.

I have similar queries to those raised for knowledge for confidence and skills.

We have defined all abbreviations used in the tables.

"There were no statistically significant differences in clinician-reported levels of confidence in working with different groups of CYP ($p = .424$)." – This doesn't follow on from the previous section.

We have redone the analysis and results.

"Therefore, there was no statistically significant difference ($p = .049$)" – What does this relate to?

We have redone the analysis and results.

Table 6 – Why do you choose to report those percentages for 2, 1 and 0 as <2%. 0 could be 0%, 1 0.9% etc?

We have corrected the %.

Could individuals only select one option? If not, how many selected multiple?

One option.

Discussion

"Although several group differences were statistically significant, statistical significance does not necessarily indicate clinical importance; rather, these findings reflect relative differences in clinician self-reported preparedness, with mean ratings generally in the moderate range (Gikandi et al., 2024)."

This is true, you need to ensure you interpret your findings clinically also.

How many individuals report training in both? How does the different uptake reflect availability in different areas? Are there any differences by type of individual / where they are based?

This has been updated based on the updated analysis and results.

Access criteria

Please revise this sentence. "In line with the current survey suggested that a higher proportion of respondents highlighted access criteria for CYP with intellectual disabilities (38.4%) than.."

Revised.

Overall, whilst I believe that your conclusions are likely to be correct, I have concerns around some of the assumptions in the statistical methods and some of the presentation. I feel that this needs to be reviewed before concluding. Have you had any conversations with a statistician regarding the most appropriate way to answer your questions and display the findings?

Thank you for your suggestion. A statistician was consulted, and they reviewed all methods, analysis, discussion and conclusion. The methods, tests, analysis, discussion and conclusion, where relevant, were updated.

Reviewer 2:

Thank you for the opportunity to review this manuscript. The paper addresses an important knowledge gap in CAMHS research, and the incorporation of PPI meaningfully strengthens the relevance of the work to affected children, young people, and families. Overall, the manuscript is well-constructed, but it would benefit from minor revisions to enhance clarity.

We appreciate your time reviewing our manuscript. After considering your feedback, we believe the updated version will meet your expectations.

1. Define autism spectrum disorder and intellectual disability to ensure consistency and accessibility for readers who may not be specialists in neurodevelopmental conditions.

Included.

2. State the regions represented in the sample and the number of respondents from each region to provide insight into potential geographic variation in clinician perspectives.

Included as part of the demographics table.

3. Specify the clinician types by region and note whether there were differences in professional backgrounds across regions, as this may influence interpretation of the findings.

Thank you for this helpful suggestion. We considered presenting clinician profession by geographical region. However, the purpose of the study was to describe clinicians' perspectives on delivering psychological therapy across mainstream CAMHS in England, rather than to compare regional or profession-specific differences. Given the modest sample sizes within individual regions and professional groups, further stratification would have resulted in very small cell counts and largely descriptive findings that were beyond the scope

of the study. We have therefore retained the overall description of clinician characteristics (and included profession and geographical representation) but have not presented profession by region, as the study was not designed or powered to investigate regional differences.

VERSION 2 - REVIEW

**Reviewer**	**3**
**Name**	**Kurth, Tobias**
**Affiliation**	**Charité Universitätsmedizin Berlin, Institute of Public Health**
**Date**	**20-Sep-2026**
**COI**	**Yes- TK reports having received research grants from the German Federal Joint Committee (G-BA) and personal compensation from the North-East German Society for Gynecological Oncology (NOGGO), AbbVie, the BMJ Group, and Frontiers Media S.A.**

In this cross-sectional survey study, Williams and colleagues examined clinicians' perspectives on delivering psychological therapy to autistic children and young people (CYP) and CYP with intellectual disabilities in mainstream CAMHS in England.

The study addresses an important topic. I understand that this is a revised manuscript and that I was not involved in the previous review. The revised paired analyses address an important concern raised previously. I nevertheless have several methodological comments that the authors may want to consider.

1. The study objectives and comparative analyses should be better aligned. Most research questions are descriptive, whereas the main inferential analyses compare ratings across autistic CYP, CYP with intellectual disabilities, and CYP without either diagnosis. The latter group is not clearly incorporated into the stated research questions. If these within-clinician comparisons are a principal objective, this should be stated explicitly and the primary outcomes and contrasts clearly defined.

2. The comparative analyses are based on selected subsets of clinicians. The Wilcoxon analysis includes only clinicians with experience of both autistic CYP and CYP with intellectual disabilities, while the Friedman analyses are restricted to clinicians with experience across all three CYP groups. The resulting comparisons therefore describe within-clinician differences among these selected respondents and should not automatically be generalized to all participants or CAMHS clinicians more broadly. This should be reflected consistently in the interpretation of the results.

3. The analysis and reporting of the ordinal survey measures could be improved. The use of Wilcoxon signed-rank and Friedman tests is reasonable for paired ordinal responses, but their justification should be based on the measurement scale and paired design rather than a Shapiro-Wilk test for normality. Medians and interquartile ranges, or full response distributions, may be more informative than means and standard deviations. In addition, effect sizes or paired differences should be reported alongside p-values, and the approach to multiplicity should be clarified for the multiple knowledge-item comparisons.

4. The conclusions should be more closely aligned with what was measured. Self-reported knowledge, confidence and skills reflect perceived preparedness rather than objectively assessed competence. The study also did not evaluate whether additional training or specific adaptations improve care, nor whether current practices lead to unmet mental health needs, disengagement, or systemic exclusion from CAMHS. These may be important hypotheses or implications for future research, but they should not be presented as conclusions arising directly from this cross-sectional clinician survey.

VERSION 2 - AUTHOR RESPONSE

Reviewer: 3
Comments to the Author:
In this cross-sectional survey study, Williams and colleagues examined clinicians' perspectives on delivering psychological therapy to autistic children and young people (CYP) and CYP with intellectual disabilities in mainstream CAMHS in England.

The study addresses an important topic. I understand that this is a revised manuscript and that I was not involved in the previous review. The revised paired analyses address an important concern raised previously. I nevertheless have several methodological comments that the authors may want to consider.
We thank the reviewer for their time; all methodological comments have been addressed, and additions reviewed by a statistician before resubmission.

1. The study objectives and comparative analyses should be better aligned. Most research questions are descriptive, whereas the main inferential analyses compare ratings across autistic CYP, CYP with intellectual disabilities, and CYP without either diagnosis. The latter group is not clearly incorporated into the stated research questions. If these within-clinician comparisons are a principal objective, this should be stated explicitly and the primary outcomes and contrasts clearly defined.

We thank the reviewer. We have revised the study objectives and research questions to explicitly state that comparisons of clinician-reported knowledge, confidence and

skills were conducted across CYP with no autism or intellectual disabilities diagnoses, autistic CYP, and CYP with intellectual disabilities. We have also clarified the primary outcomes and planned comparisons in the Methods section.

2. The comparative analyses are based on selected subsets of clinicians. The Wilcoxon analysis includes only clinicians with experience of both autistic CYP and CYP with intellectual disabilities, while the Friedman analyses are restricted to clinicians with experience across all three CYP groups. The resulting comparisons therefore describe within-clinician differences among these selected respondents and should not automatically be generalized to all participants or CAMHS clinicians more broadly. This should be reflected consistently in the interpretation of the results.

We agree and have revised the Methods, Results, Discussion and Conclusion to make explicit that inferential analyses were restricted to clinicians with relevant experience of the CYP groups being compared. Interpretations have been revised accordingly and no longer imply generalisation to all CAMHS clinicians.

3. The analysis and reporting of the ordinal survey measures could be improved. The use of Wilcoxon signed-rank and Friedman tests is reasonable for paired ordinal responses, but their justification should be based on the measurement scale and paired design rather than a Shapiro-Wilk test for normality. Medians and interquartile ranges, or full response distributions, may be more informative than means and standard deviations. In addition, effect sizes or paired differences should be reported alongside p-values, and the approach to multiplicity should be clarified for the multiple knowledge-item comparisons.

Thank you for your comment. The rationale for using the Wilcoxon signed-rank test and the Friedman test was described in the analysis methods section. Description of the effect sizes for the Wilcoxon signed-rank test and the Friedman test was added in the analysis methods section, and the results of effect sizes were included in the relevant results section. Medians (Mdn) and interquartile ranges (IQR) were added in the results section. Bonferroni's method was used to address multiplicity and is explained in the analysis methods section.

4. The conclusions should be more closely aligned with what was measured. Self-reported knowledge, confidence and skills reflect perceived preparedness rather than objectively assessed competence. The study also did not evaluate whether additional training or specific adaptations improve care, nor whether current practices lead to unmet mental health needs, disengagement, or systemic exclusion from CAMHS. These may be important hypotheses or implications for future research, but they should not be presented as conclusions arising directly from this cross-sectional clinician survey.

Thank you for this important comment. We agree that the study assessed clinicians' self-reported knowledge, confidence, and skills, which reflect perceived

preparedness rather than objectively measured competence. We have therefore revised the Discussion and Conclusion to consistently refer to self-reported knowledge, confidence, skills, and preparedness. We have also removed causal statements regarding unmet mental health needs, disengagement from services, and systemic exclusion, as these outcomes were not directly assessed in the current study. Statements regarding training needs, service access, and treatment adaptations have been reframed as implications for consideration and areas for future research rather than conclusions arising directly from the survey findings.

Reviewer: 3
If you have selected ‘Yes’ above, please provide details of any competing interests.:
TK reports having received research grants from the German Federal Joint Committee (G-BA) and personal compensation from the North-East German Society for Gynecological Oncology (NOGGO), AbbVie, the BMJ Group, and Frontiers Media S.A.
